# Linking a polyketide synthase gene cluster to 6-pentyl-alpha-pyrone, a *Trichoderma* metabolite with diverse bioactivities

**DOI:** 10.1186/s12934-025-02718-9

**Published:** 2025-04-21

**Authors:** Daniel Flatschacher, Alexander Eschlböck, Siebe Pierson, Ulrike Schreiner, Valentina Stock, Arne Schiller, David Ruso, Maria Doppler, Veronika Ruzsanyi, Mario Gründlinger, Christoph Büschl, Rainer Schuhmacher, Susanne Zeilinger

**Affiliations:** 1 Department of Microbiology, University of Innsbruck, Technikerstrasse 25, Innsbruck, 6020 Austria; 2 Institute for Breath Research, University of Innsbruck, Innsbruck, Austria; 3 Core Facility Bioactive Molecules: Screening and Analysis, University of Natural Resources and Life Sciences, Tulln, Austria; 4 Institute of Bioanalytics and Agro-Metabolomics, Department of Agrobiotechnology (IFA-Tulln), University of Natural Resources and Life Sciences, Tulln, Austria

**Keywords:** *Trichoderma atroviride*, 6-pentyl-alpha-pyrone, Polyketide synthase, Biocontrol, Biosynthetic gene cluster, Specialized metabolites

## Abstract

**Background:**

Members of the fungal genus *Trichoderma* are well-known for their mycoparasitic and plant protecting activities, rendering them important biocontrol agents. One of the most significant specialized metabolites (SMs) produced by various *Trichoderma* species is the unsaturated lactone 6-pentyl-alpha-pyrone (6-PP). Although first identified more than 50 years ago and having pronounced antifungal and plant growth-promoting properties, the biosynthetic pathway of 6-PP still remains unresolved.

**Results:**

Here, we demonstrate that 6-PP is biosynthesized via the polyketide biosynthesis pathway. We identified Pks1, an iterative type I polyketide synthase, as crucial for its biosynthesis in *Trichoderma atroviride*, a species recognized for its prominent 6-PP production abilities. Phylogenetic and comparative genomic analyses revealed that the *pks1* gene is part of a biosynthetic gene cluster conserved in those *Trichoderma* species that are known to produce 6-PP. Deletion of *pks1* caused a complete loss of 6-PP production in *T. atroviride* and a significant reduction in antifungal activity against *Botrytis cinerea* and *Rhizoctonia solani*. Surprisingly, the absence of *pks1* led to enhanced lateral root formation in *Arabidopsis thaliana* during interaction with *T. atroviride*. Transcriptomic analysis revealed co-regulation of *pks1* with adjacent genes, including candidates coding for a C3H1-type zinc finger protein and lytic polysaccharide monooxygenase, suggesting coordination between 6-PP biosynthesis and environmental response mechanisms.

**Conclusion:**

Our findings establish *pks1* as an essential gene for 6-PP biosynthesis in *T. atroviride*, providing novel insights into the production of one of the most significant compounds of this mycoparasite. These findings may pave the way for the development of improved biocontrol agents and the application of 6-PP as potent biopesticide contributing to an eco-friendly and sustainable way of plant disease management.

**Supplementary Information:**

The online version contains supplementary material available at 10.1186/s12934-025-02718-9.

## Background

Biocontrol, the strategic use of organisms to reduce and manage pest populations, is increasingly being recognized as a crucial tool in sustainable agriculture and disease management. Fungi of the genus *Trichoderma* are widely recognized for their biocontrol properties. They are not only capable of inhibiting the growth of various plant pathogenic fungi through mycoparasitism and the production of antifungal compounds but also promote plant growth, vigor, and tolerance to biotic and abiotic stresses through the secretion of plant growth-promoting hormones and elicitors [1]. The versatile lifestyles of *Trichoderma* spp. allow them to inhabit a wide range of ecological niches, from soil to the rhizosphere, and even the endosphere of plants. As many *Trichoderma* spp. are opportunistic and avirulent plant symbionts, they have evolved numerous strategies to establish mutually beneficial relationships with plants. These strategies include induction of systemic resistance, competition with plant pathogenic microorganisms for nutrients and space, parasitism of plant pathogens, and modulation of the plant immune system [2]. *Trichoderma* fungi have also been extensively studied for their ability to produce an array of diverse specialized metabolites (SMs), including non-ribosomal peptides (NRPs) such as trichorzianines or trichoatrokontins, terpenoids such as trichodermin, and polyketides (PKs), all of which exhibit broad bioactive properties [3–5].

**Fig. 1:**
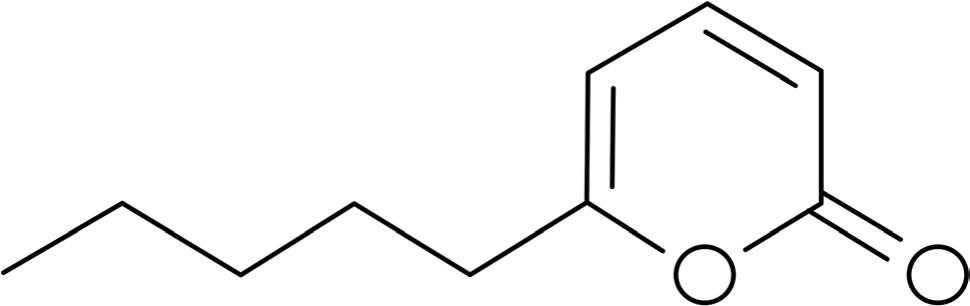
Structure of 6-pentyl-alpha-pyrone (6-PP)

One of the most significant and well-documented SMs produced by certain *Trichoderma* species, such as *Trichoderma atroviride*, is 6-pentyl-alpha-pyrone (6-PP; Fig. 1) [6]. This compound has been extensively studied for its diverse bioactivities and its role in fungal interactions. One of the key characteristics of 6-PP is its antifungal property, as it was shown to inhibit the growth of several plant pathogenic fungi, including *Botrytis cinerea*, *Fusarium oxysporum*, and *Rhizoctonia solani* [7, 8]. Studies have shown a positive correlation between *Trichoderma* strains that produce high concentrations of 6-PP and their antagonistic ability, with *T. atroviride* being the most efficient producer [9–12]. Beyond its antifungal properties, 6-PP has been found to promote plant growth and to alter root architecture, including inhibition of primary root growth and induction of lateral root development [13]. These alterations in root architecture can enhance the plant’s ability to absorb nutrients and water, thereby promoting overall plant health and productivity [14].

6-PP can easily diffuse within the environment, probably also acting as signaling molecule that facilitates communication both within and between organisms. Moreno-Ruiz et al. [15] revealed a role of 6-PP as endogenous self-signaling compound. It elicited strong chemotropic responses in conidial germlings of *T. atroviride* and at later developmental stages led to an increase in hyphal density. 6-PP may also be crucial for stimulating cell fusion in *T. atroviride*, which is essential for effective mycoparasitism [15, 16]. These effects, together with its volatility, are also supposed to impact plant-fungi interactions, with 6-PP acting as priming or protection factor that enhances the plant’s resistance to pathogens [17]. Recent studies have further revealed the potential of 6-PP to inhibit the replication of canine coronaviruses, highlighting its broader applications beyond its agricultural benefits [18].

Despite the growing interest in *Trichoderma*-mediated biocontrol and the multifaceted biological effects of 6-PP, the molecular pathway underlying its biosynthesis has not yet been elucidated. Early experiments based on isotopic labeling suggested that the beta-oxidation of linoleic acid by lipoxygenase is a main step in the biosynthetic pathway of 6-PP production [19]. However, deletion of the single lipoxygenase-encoding gene *lox1* encoded in the *T. atroviride* genome did not affect 6-PP production, indicating that the biosynthetic pathway of 6-PP may involve other enzymes or metabolic processes [20]. Deletion of *tmk1*, encoding a mitogen-activated protein kinase, in *T. atroviride* resulted in reduced expression of *lox1*, yet led to an overproduction of 6-PP [20, 21]. This suggests a repressing effect of Tmk1 on the metabolic pathway of 6-PP biosynthesis.

Environmental factors such as light, temperature, and pH modulate SM production in fungi [22]. Accordingly, 6-PP biosynthesis in *T. atroviride* is significantly influenced by light, with highest levels being produced under dark cultivation conditions, while exposure to light in the form of light-dark cycles significantly reduced its biosynthesis [23, 24].

It is generally accepted that most compounds containing an α-pyrone moiety are biosynthesized via the polyketide pathway [25]. Biosynthetic enzymes for fungal polyketide production often are encoded in biosynthetic gene clusters (BGCs) in the genome. These BGCs consist of tightly linked sets of genes that participate in a common, discrete metabolic pathway. The genes are in physical proximity to each other in the genome, and their expression is often co-regulated [26]. Polyketide biosynthesis shares many similarities with fatty acid biosynthesis, with similar mechanisms of chain elongation and the use of simple building blocks such as acetyl-CoA and malonyl-CoA [27]. The core enzymes involved in polyketide biosynthesis are type I, type II and type III polyketide synthases (PKSs) [28], whereas most fungal PKSs are of type I [29]. Fungal type I PKSs are monomodular enzymes, containing several domains that function iteratively. This iterative function allows them to repeatedly catalyse specific modifications during the stepwise biosynthesis of the polyketide product [30].

Fungal type I PKSs can be further classified into highly-reducing (HR-), partially-reducing (PR-) and non-reducing (NR-) PKSs based on their domain composition. HR-PKSs contain enoyl-reductase (ER), dehydratase (DH), and ketoreductase (KR) domains. In contrast, NR-PKSs lack these three domains and contain only ketoacyl-synthase (KS), acyl transferase (AT), and acyl carrier protein (ACP) domains. PR-PKSs fall between these two classes, as they lack some of the reducing domains, leading to partially reduced intermediates [29, 30].

The PKS type also influences the nature of the polyketides produced (Chooi and Tang, 2012). NR-PKSs are primarily responsible for synthesizing cyclic, aromatic polyketides. Aromatic polyketides are a class of compounds containing at least one aromatic ring. Well-known aromatic polyketides synthesized by NR-PKSs are anthraquinones [31]. Anthraquinones have been studied for their potential medicinal properties, including their use as immunostimulants and their potential anti-cancer effects [32]. On the other hand, several HR-PKSs are involved in the production of cyclic nonaromatic polyketides such as lovastatin, a compound that is used as a cholesterol-lowering drug [33].

Given the significant role of 6-PP in *T. atroviride* and in its interactions with host fungi and plants, including potential applications in sustainable agriculture, it is crucial to understand the molecular mechanisms underlying the biosynthesis of this important metabolite. In this study, we demonstrate that 6-PP is biosynthesized via the polyketide biosynthesis pathway. We identified and analyzed the responsible gene cluster in the *T. atroviride* genome and functionally characterized the PKS-encoding core gene essential for 6-PP biosynthesis.

## Methods

### Fungal strains and culture conditions

The wild type (WT) strain *Trichoderma atroviride* P1 (ATCC 74058; Ascomycota) was used as the parental strain for the generation of the deletion mutants *∆pks1A*, *∆pks1B*, and *∆pks1C*. For confrontation and inhibition assays, the plant-pathogens *Rhizoctonia solani* (Basidiomycota; pathogenic isolate obtained from the collection of the Institute of Plant Pathology, Università degli Studi di Napoli “Federico II,” Naples, Italy) and *Botrytis cinerea* B05.10 (Ascomycota) were used as host fungi.

*T. atroviride* was cultured on potato dextrose agar (PDA; Becton, Dickinson and Company, Le Pont De Claix, France). Hygromycin B (200 µg/mL; Calbiochem, Merck KGaA, Darmstadt, Germany) was used for mutant selection. If not stated otherwise, three biological replicates of the WT and *pks1* deletion mutants (*∆pks1A*, *∆pks1B*, and *∆pks1C*) were processed in all phenotypic characterization experiments. As all three deletion mutants showed reproducible and comparable phenotypes and characteristics in growth assays, *∆pks1A* was randomly selected for all subsequent experiments. Fungal cultures were incubated at 25 °C under either light-dark (12:12 h cycle, 20 µmol m^− 2^ s^− 1^; Snijders Micro Clima-Series TM Labs Economic Lux Chamber; Snijders Labs, Tilburg, Netherlands) or reduced light conditions, as described in Missbach et al. [24].

### Sequence analysis and gene cluster comparison

The *T. atroviride* P1 genome was screened using antiSMASH [34] to identify scaffolds/contigs encoding enzymes involved in the biosynthesis of SMs. Scaffold 9:1-67402 was investigated further using the Joint Genome Institute database (JGI, https://mycocosm.jgi.doe.gov/Triatrov1/Triatrov1.home.html [35]), to refine gene boundaries and introns. UniProt [36] was used to identify protein domains and similarities to proteins from other *Trichoderma* species. Comparative genomics/clinker analysis was performed using Cagecat [37] and the genomes of *Trichoderma gamsii*, *Trichoderma asperellum*, and *T. atroviride* IMI206040 were retrieved from the National Center for Biotechnology Information (NCBI). Sequence alignments and phylogenetic trees were generated with Muscle and the neighbor-joining method using 1000 bootstrap replications in MEGA 11 [38]. The phylogenetic tree is based on the complete protein sequence of *T. atroviride* P1 protein ID 453700 (Pks1) and PKS sequences from various fungi producing known pyrones (*Alternaria* spp., *Aspergillus* spp., *Calcarisporium arbuscula*, *Penicillium polonicum*), flavonoids (*Aspergillus candidus*, *Penicillium expansum*, *Pestablotiopsis fici*), anthraquinones (*Aspergillus* spp., *Fusarium* spp., *Metarhizium album*, *Pseudocercospora fijiensis*, *Trichoderma reesei*), and macrolides (*Fusarium graminearum*, *Stachybotrys chartarum*). All protein sequences were retrieved from UniProt. Interactive Tree Of Life (iTOL) was used for visualizing and editing the phylogenetic tree [39].

### Generation and validation of *pks1* gene deletion mutants

A plasmid bearing the *pks1* deletion construct was generated by assembling the following PCR amplified fragments with NEBuilder HiFi DNA Assembly Kit (New England Biolabs, Germany): a hygromycin B resistance-conferring selection cassette derived from plasmid pGFP-XYR1 [40] (amplified with primers 453700_hph_F and 453700_hph_R); upstream and downstream flanking regions (approximately 1 kb each) of the *pks1* locus, which were obtained from *T. atroviride* WT genomic DNA (using primers 453700_5-flank_F and 453700_5-flank_R for the 5’ flank and 453700_3-flank_F and 453700_3-flank_R for the 3’ flank); and the backbone from the pGFP_XYR1 plasmid (amplified with primers 453700_backbone_F and 453700_backbone_R). The integrity of the assembled plasmid was verified by PCR using primers 453700_5-flank_F and 453700_3-flank_R and restriction digestion using *Sal*I. The selection cassette containing the *Escherichia coli hph* gene, which confers resistance to hygromycin B, was split into 5′ (amplified with primers 453700_5-flank_F and Catellet HY-R) and 3′ (amplified with primers Catellet YG-F and 453700_3-flank_R) split marker fragments as previously described [41]. These fragments were used for homologous recombination via the split marker approach [42]. All fragments were purified using the Monarch PCR & DNA Cleanup Kit (New England Biolabs Inc., Ipswich, MA, USA), following the manufacturer’s instructions.

CRISPR/Cas9 target sequences (crRNA) for the *pks1* gene locus were designed using the chop-chop tool [43]. After selecting the crRNA sequences with optimal locations and predicted high activity scores, a BLAST search against the *T. atroviride* P1 genome database on JGI was performed to ensure minimal off-target scores. The designed crRNAs (453700_5-flank_crRNA and 453700_3-flank_crRNA), tracrRNA and the Cas9 endonuclease were purchased from Integrated DNA Technologies (IDT, Coralville, Iowa, USA). The preparation of sgRNA and the RNP complex was carried out according to Tannous et al. [44] and Luo et al. [45]. Protoplast generation and PEG-mediated transformation of the split-marker constructs and the CRISPR-Cas9 RNP complex were performed as previously described [46].

The resulting transformants were subjected to three rounds of single-spore isolation on selective media containing 200 µg/mL hygromycin B (Calbiochem, Merck KGaA, Darmstadt, Germany) to ensure mitotic stability. Homologous recombination at the target locus and successful gene deletion were confirmed in three independent and mitotically stable deletion mutants (*∆pks1A*, *∆pks1B*, and *∆pks1C*) via PCR using the primer pairs 453700_locus-5_F + 453700_locus-5_R and 453700_locus-3_F + 453700_locus-3_R, binding the genomic DNA regions flanking the integration sites, and primer pairs 453700_locus-5_F + Pgapdh-hph-R and Pgapdh-hph-F + 453700_locus-3_R, binding inside the selection cassette. All primer sequences are listed in supplementary Table S1. The PCR-based genotyping strategy and results of genotyping of the *∆pks1A*, *∆pks1B*, and *∆pks1C* mutants are shown in supplementary Fig. S1.

### Growth assay and Inhibition assessment

*T. atroviride* WT and its *pks1* deletion mutants were grown on PDA at 25 °C under light-dark cycles (12:12 h) for 6 days. The colony diameters (d) were measured after 24, 48, and 72 h of growth, and the radial growth rate (cm/d) was calculated. Photos were taken daily to visually capture any changes. All assays were performed in triplicates.

To assess the inhibitory effect of soluble, diffusible metabolites secreted by the *T. atroviride* WT and the *∆pks1*A deletion mutant, inhibition assays were conducted using *B. cinerea* and *R. solani* as test fungi. A mycelia-covered plug (6 mm diameter) of pre-cultures of either the WT or the deletion mutant was placed upside down in the center of a fresh PDA plate covered with a cellophane membrane and cultivated for 2 days at 25 °C under reduced light conditions. The area covered by the colony was measured and the cellophane carrying the *T. atroviride* colony was removed. For *B. cinerea*, 100 µL of a spore suspension containing 1 × 10^6^ spores/mL were spread over the surface of each plate. For *R. solani*, mycelia-covered plugs (6 mm diameter) taken from the actively growing colony margins of a pre-culture were transferred to the center of the test plates. The inoculated plates were further incubated for 5 days at 23 °C under reduced light conditions. The diameters of the inhibition zones in the developing *B. cinerea* (B) cultures were measured and calculated as an index (%) representing growth inhibition compared to former *T. atroviride* (T) colony size (dT/dB). Rather than measuring inhibition zones as with *B. cinerea*, the growth of *R. solani* colonies was monitored. The diameters of *R. solani* colonies were measured and used to calculate the inhibition index (dT/(dT + dR), in %). All assays were performed in triplicates.

### Interaction assays

Mycelia-covered plugs (6 mm diameter) from the actively growing colony margins of pre-cultures of the WT, *∆pks1A*, and host fungi (*B. cinerea*, *R. solani*) were collected. Both, the WT and *∆pks1A* were confronted with a host fungus. To account for different growth rates, *R. solani* was inoculated two days prior to the introduction of the *T. atroviride* WT strain. In addition to confrontations with host fungi, each strain was grown alone and in self-confrontation. The plates were incubated at 25 °C under reduced light conditions. The progress of the mycoparasitic interaction was monitored and photographically documented. All interaction assays were performed in triplicates.

### Transcriptomic analysis

Cultures for transcript analysis were cultivated for 72 h at 25 °C on PDA plates covered with cellophane under light-dark and reduced light conditions. RNA extraction and RT-qPCR were performed as previously described [47]. Total RNA from the harvested biomass was extracted using TRIzol Reagent (Invitrogen, Karlsruhe, Germany) and reverse transcribed to cDNA using the Revert Aid H- First Strand cDNA Synthesis Kit (ThermoFisher Scientific Baltic UAB, Vilnius, Lithuania). RiboLock RNase Inhibitor (ThermoFisher Scientific Baltic UAB, Vilnius, Lithuania) was added according to the manufacturer’s instructions. RT-qPCR was performed using the LUNA Universal qPCR Master Mix (New England BioLabs GmbH) in a qTOWER3 G cycler (Analytik Jena AG, Jena, Germany) with the primers given in supplementary Table S2. Expression levels were normalized by using *tbp* and *vma* (supplementary Table S2) as reference genes and relative quantity was calculated using qRAT [48].

### Specialized metabolite analysis

Cultures for metabolite extraction were incubated for 96 h at 25 °C under reduced light conditions on PDA plates covered with cellophane. The cellophane carrying the fungal mycelia was removed and agar pieces were excised following a defined spatial pattern to represent the entire plate. In total, approximately 1 g of agar was collected from each plate in three independent biological replicates.

For high-performance thin-layer chromatography (HPTLC) analysis, metabolites were extracted from the collected agar pieces by addition of 5 mL 50% acetone in water and supersonication in an ultrasonic bath for 15 min. Thereafter, 4 mL ethyl acetate (EtOAc) were added, the tube was vortexed and centrifuged at 3.000 x g for 1 min for phase separation. The collected organic phase was evaporated to dryness, re-collected in 120 µL methanol (MeOH), and 20 µL were used for HPTLC separation and analysis. Samples were spotted on a silica gel plate (HPTLC silica gel 60 F254S, Merck KGaA, Darmstadt, Germany) with an automatic TLC sampler (ATS 4, CAMAG, Muttenz, Switzerland) and separated with diethyl ether: petroleum ether 4.5:5.5 (*v*/*v*) as the mobile phase. Plates were developed in an automated developing chamber (ADC2, CAMAG, Muttenz, Switzerland) at a relative humidity of 11% and a migration distance of 80 mm. Pictures were taken at 254 nm with a TLC visualizer (CAMAG, Muttenz, Switzerland) using the software visionCATS 4.0 (CAMAG, Muttenz, Switzerland). Two µg of an analytical-grade standard of 6-PP (≥ 96%; Sigma-Aldrich, Vienna, Austria) were applied to the HPTLC and chromatographed as reference.

For liquid-chromatography high-resolution mass spectrometry (LC-HRMS), fungal agar plugs of the *Δpks1A* mutant and the WT strain were extracted as described above for HPTLC analysis, and measured together with an authentic 6-PP reference standard (Sigma-Aldrich, Vienna, Austria) on a Vanquish Horizon UHPLC system (Thermo Fisher Scientific, Waltham, MA, USA) coupled via a Heated Electro Spray Ionization (HESI) source to a QExactive HF Orbitrap system (Thermo Fisher Scientific, Bremen, Germany). The LC autosampler was cooled to 10 °C and the column oven was heated to 25 °C. 2µL of the standard and the fungal extracts were injected onto a Waters XBridge C18 column (3.5 μm, 2.1 × 150 mm; Waters, Milford, MA, USA). Water (A) and methanol (B), both with 0.1% formic acid were used as eluents for gradient elution at a constant flow rate of 250 µL/min. After an initial 1-minute hold time at 10% B, eluent B was increased to 100% over 9 min, followed by 3 min hold at 100% B. Finally, the column was re-equilibrated at 10% B for another 7 min. LC-HRMS full scan and MS/MS measurements were performed with a scan range of *m/z* 60 to 500 in fast polarity switching mode and a resolving power setting of 120,000 full width at half maximum (FHWM) at *m/z* 200. The spray voltage was adjusted to 3.5 kV for positive and − 3 kV for negative ionization mode. Sheath and auxiliary gas flow rates were set to 55 and 5 arbitrary units, respectively, and the vaporizer temperature was maintained at 350 °C. MS/MS fragment spectra were recorded in data dependent MS/MS mode (Top 5) in positive ionization mode with stepped collision energies of 20, 45 and 70 eV and a resolving power setting of 30.000 FWHM at *m/z* 200 using an inclusion list. Data evaluation was carried out with FreeStyle (v1.8, Thermo Fisher Scientific) and SIRIUS (v5.8.0 [49]). Raw-LC-HRMS/MS data were processed with MZmine (v4.5.20 [50]), using the provided mzwizard for initial batch queue design. Additional steps included the compound-annotation with predicted structurally similar compounds and selection of only such annotated compounds. Furthermore, the molecular networking parameters of the queue were adapted and a cosine-cutoff-score of 0.6 was used (instead of the default value of 0.7). The generated feature-based molecular network was then further illustrated and processed with Cytoscape (v3.9.1 [51]).

### Plant interaction assay

The *Arabidopsis thaliana* – *T. atroviride* co-cultivation experiment was performed in agar plates containing PNM9 medium, prepared by adding 0.5% glucose to PNM [52]. *A. thaliana* Col-0 seeds were sterilized using a 50% bleach solution [53], stratified for 3 days at 4 °C and then incubated at 22 °C ± 1 °C with a 16 h-light/8 h-dark cycle and light-intensity of 80–100 µmol m^− 2^ s^− 1^. Per plate, nine seeds were evenly spaced in a line and plates were wrapped with Parafilm. Plates were incubated vertically at a 65° angle to allow root growth along the agar surface. After 5 days of incubation, 1 × 10^6^ spores of *T. atroviride* WT or Δ*pks1*A were spotted at a distance of 5 cm from the primary root tip. Sterile distilled water was added to the control plates. Plates were incubated for another 5 days, after which the plants and fungal colony were phenotypically assessed. The lateral roots were counted and the fresh weight, primary root length as well as the radius of the fungal colony were measured. Results were validated with an ANOVA statistical analysis. Post hoc t-test with Bonferroni-correction was used to assess the significance of differences in plant growth and root system architecture between treatments.

## Results

### Comparative analyses of Pks1 and its biosynthetic gene cluster

**Fig. 2:**
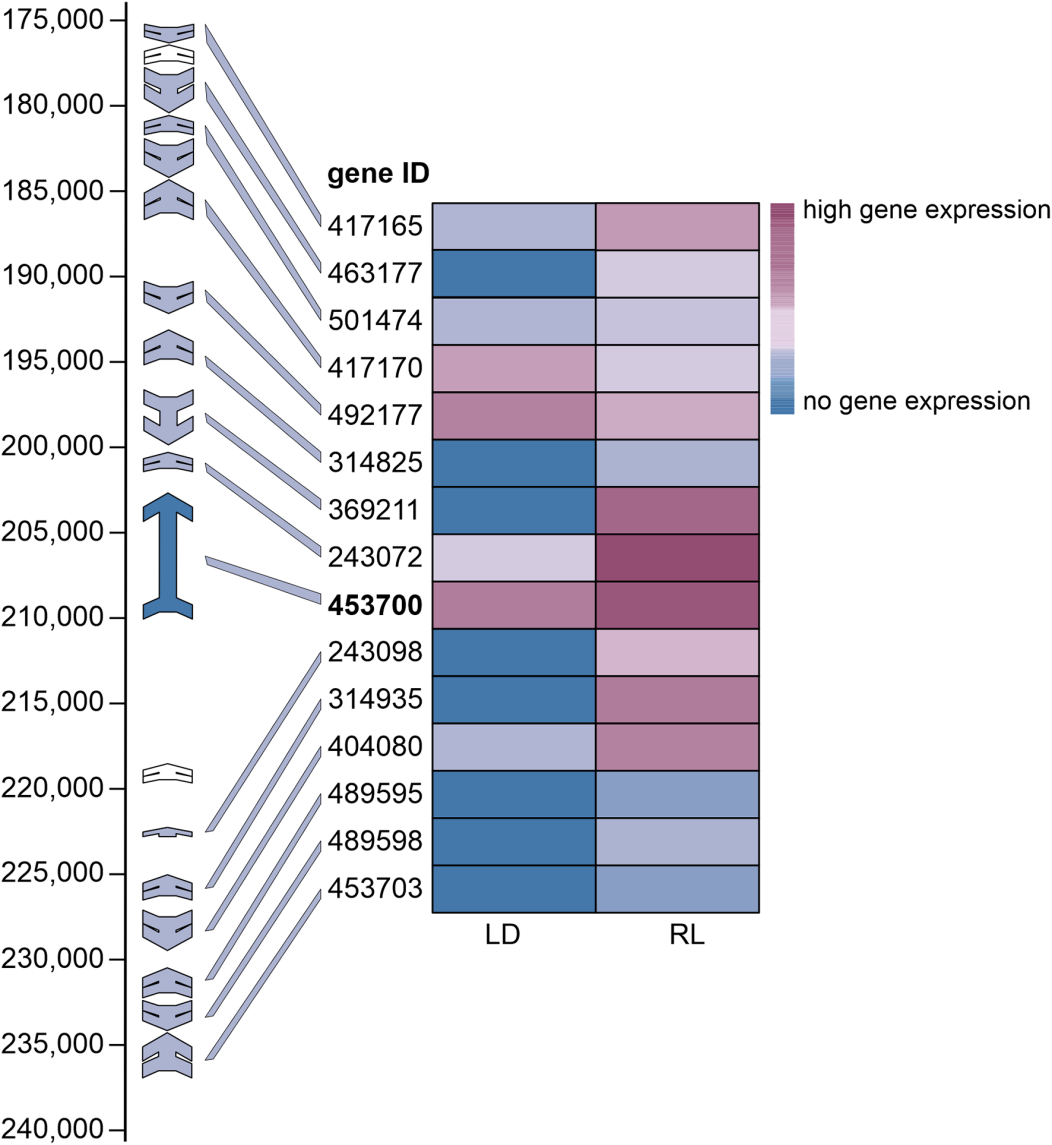
Expression analysis of the putative members of the predicted *pks1* gene cluster in *T. atroviride* P1 grown under light-dark or reduced light conditions. The schematic representation on the left illustrates the genomic organization of the BGC in *T. atroviride* P1. Each arrow denotes a gene within the cluster, with the gene identifier given next to each arrow. The heatmap depicts the relative mRNA levels of each gene under the two different light conditions tested: light-dark cycle (LD) and reduced light (RL). The color represents the relative mRNA quantity normalized to two internal reference genes

We have previously shown that 6-PP is produced by *T. atroviride* in a light-dependent manner [23]. Due to its high expression upon fungal growth in reduced light conditions (Fig. 2), *pks1* (ID 453700) was selected for further characterization as a candidate putatively involved in the biosynthesis of 6-PP.

**Fig. 3:**
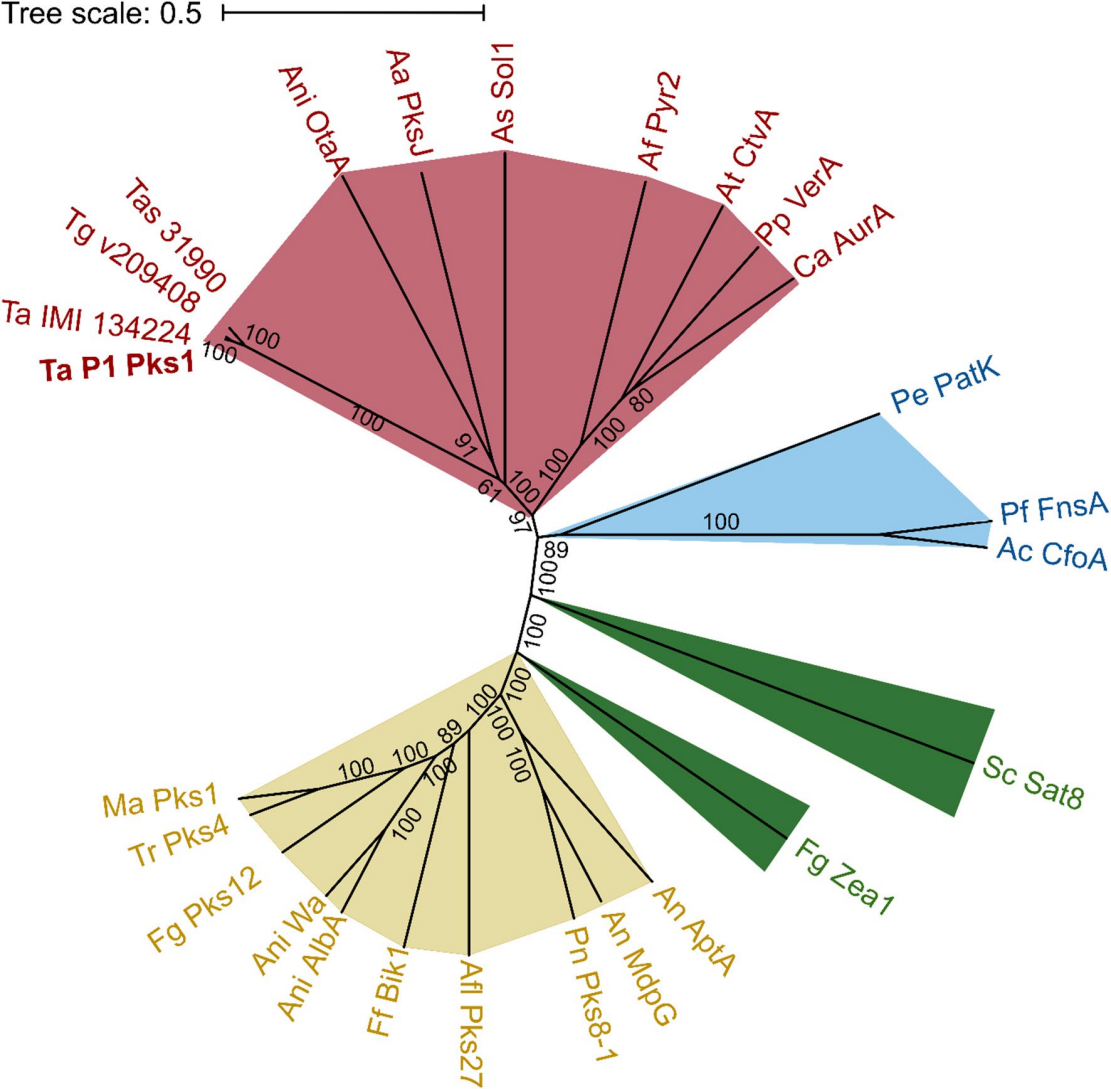
Analysis of phylogenetic relationship of *T. atroviride* Pks1 The phylogenetic tree was generated by comparing amino acid sequences of PKSs associated with known compound classes from other fungi to *Trichoderma* Pks1 orthologues. Neighbor-joining algorithm of MEGA11 was used. Numbers indicate the bootstrap probability values of observing the branch topology shown. Scale bar indicates substitutions per site. Proteins are color-coded according to their association with biosynthetic pathways of specific compound classes: yellow for anthraquinone biosynthesis, green for macrolide biosynthesis, blue for flavonoid biosynthesis, and red for pyrone biosynthesis. The UniProt database was used to retrieve protein names. Pks1 of *T. atroviride* is highlighted in bold. Organism abbreviations correspond to specific fungal species, followed by the respective protein name: Aa (*Alternaria alternata*) – PksJ (Polyketide synthase PksJ); Ac (*Aspergillus candidus*) – CfoA (Chalcone synthase CfoA); Af (*Aspergillus fumigatus*) – Pyr2 (Non-reducing polyketide synthase Pyr2); Afl (*Aspergillus flavus*) – Pks27 (Non-reducing polyketide synthase Pks27). An (*Aspergillus nidulans*) – AptA (Non-reducing polyketide synthase AptA), MdpG (Atrochrysone carboxylic acid synthase); Ani (*Aspergillus niger*) – OtaA (Ornithine aminotransferase), AlbA (Non-reducing polyketide synthase AlbA), Wa (Conidial yellow pigment biosynthesis polyketide synthase); As (*Alternaria solani*) – Sol1 (Prosolanapyrone synthase); At (*Aspergillus terreus*) – CtvA (Highly reducing polyketide synthase CtvA); Ca (*Calcarisporium arbuscula*) – AurA (Highly reducing polyketide synthase AurA); Ff (*Fusarium fujikuroi*) – Bik1 (Bikaverin polyketide synthase Bik1); Fg (*Fusarium graminearum*) – Zea1 (Non-reducing polyketide synthase ZEA1), Pks12 (Non-reducing polyketide synthase PKS12); Ma (*Metarhizium album*) – Pks1 (Polyketide synthase 1); Sc (*Stachybotrys chartarum*) – Sat8 (Non-reducing polyketide synthase SAT8); Ta (*Trichoderma atroviride*) – Pks1; Tas (*Trichoderma asperellum*) – Pks1; Tg (*Trichoderma gamsii*) – Pks1; Tr (*Trichoderma reesei*) – Pks4 (Predicted protein, involved in green pigment biosynthesis [58]); Pe (*Penicillium expansum*) – PatK (6-Methylsalicyclic acid synthase); Pf (*Pestalotiopsis fici*) – FnsA (Naringenin synthase); Pn (*Pseudocercospora fijiensis*) – Pks8-1 (Non-reducing polyketide synthase PKS8-1); Pp (*Penicillium polonicum*) – VerA (Highly reducing polyketide synthase VerA).

To get information towards the structure of the polyketide product synthesized by Pks1, we performed a phylogenetic analysis comparing Pks1 with other PKS proteins from various fungi that have previously been associated with characterized compound classes (Fig. 3). Our analysis revealed a distinct clustering pattern, indicating a strong similarity between *T. atroviride* Pks1 and PKSs from other fungi linked to pyrone-containing compounds. Specifically, we observed that Pks1 clustered closely with PKSs from *Alternaria* spp., responsible for the biosynthesis of alternariol [54] and solanapyrone [55], as well as PKSs from *Aspergillus* spp. involved in the production of citreoviridin [56] and pyripyropene A [57] (Fig. 3, red clade). Additionally, another clade adjacent to the “pyrone” group emerged which included PKSs associated with flavonoid biosynthesis (Fig. 3, blue clade). A distantly related clade comprised of PKSs known to synthesize anthraquinones and related derivatives such as Pks4 from *T. reesei* and MdpG from *A. nidulans*, involved in monodictyphenone biosynthesis, was further noted (Fig. 3, yellow clade).

**Fig. 4:**
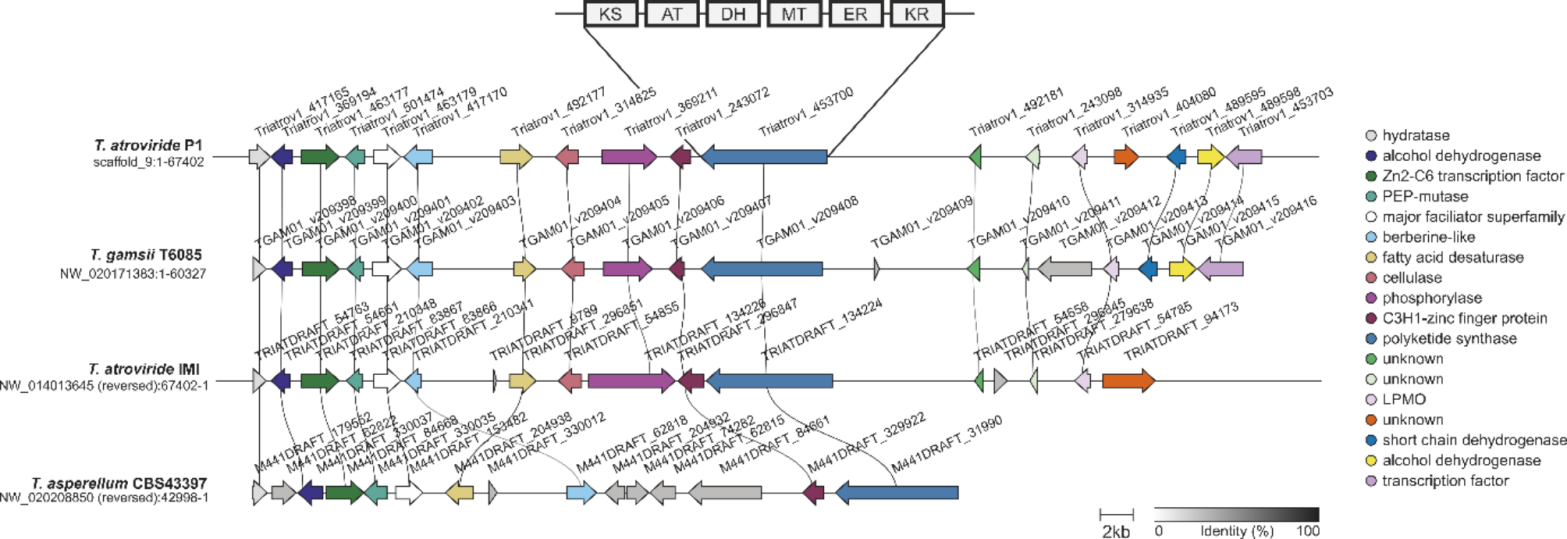
Overview of the architecture of the *pks1* gene cluster in *T. atroviride* P1 and comparison between selected 6-PP producing *Trichoderma* species. Cluster comparison based on sequence similarity and gene functional prediction. The polyketide synthase gene *pks1* in *T. atroviride* P1 and orthologs in *T. atroviride* IMI206040, *T. gamsii*, and *T. asperellum* are colored in blue. Gene orthologs in the BGC are connected by black lines and color-coded according to their predicted gene function. Schematic domain organization of Pks1 is given on top. Pks1 harbors a ketosynthase (KS), acyltransferase (AT), dehydratase (DH), methyltransferase (MT), enoyl reductase (ER), and ketoreductase (KR) domain

Given the similar architecture of fungal PKSs, the domain structure of *T. atroviride* Pks1 was analyzed using multiple domain prediction pipelines, including UniProt and SMART with standard parameters. Conserved domains typical for iterative type I PKSs, such as KR, DH and ER, were identified in addition to KS and AT domains (Fig. 4). This domain arrangement, and the absence of a thioesterase (TE) domain is characteristic for highly reducing type I PKSs that generate pyrone structures through spontaneous cyclization [59]. The close phylogenetic clustering of Pks1 with PKSs known to produce pyrone compounds (Fig. 2) further supports its likely involvement in 6-PP biosynthesis.

AntiSMASH analysis revealed that *pks1* is part of a putative BGC spanning a 67.4 kb long region in the *T. atroviride* P1 genome. Besides the gene coding for the Pks1 key enzyme, the predicted cluster contains 17 genes encoding proteins such as putative phosphorylase, hydratase, fatty acid desaturase, and two dehydrogenases. In addition, three genes encoding transcription factors are part of the predicted BGC (Fig. 4). A complete list of the predicted cluster genes along with their locus tag and putative function is given in Table 1.

**Table 1:** Genes and their putative function in the predicted *T. atroviride pks1* Triatrov1_453700 cluster

Gene Identifier	Length		Putative Function
	nt	aa	
jgi.p_Triatrov1_417165	735	244	hydratase
jgi.p_Triatrov1_369194	1074	357	alcohol dehydrogenase
jgi.p_Triatrov1_463177	1884	627	Zn2-C6 transcription factor
jgi.p_Triatrov1_501474	945	314	PEP-mutase
jgi.p_Triatrov1_463179	1410	469	major faciliator superfamily
jgi.p_Triatrov1_417170	1593	530	berberine bridge enzyme-like
jgi.p_Triatrov1_492177	1173	390	fatty acid desaturase
jgi.p_Triatrov1_314825	1275	424	cellulase
jgi.p_Triatrov1_369211	2925	974	phosphorylase
jgi.p_Triatrov1_243072	942	313	C3H1-zinc finger protein
jgi.p_Triatrov1_453700	7020	2339	**polyketide synthase**
jgi.p_Triatrov1_492181	465	154	unknown
jgi.p_Triatrov1_243098	330	109	unknown
jgi.p_Triatrov1_314935	756	251	lytic polysaccharide monooxygenase
jgi.p_Triatrov1_404080	1410	469	unknown
jgi.p_Triatrov1_489595	984	327	short chain dehydrogenase
jgi.p_Triatrov1_489598	1086	361	alcohol dehydrogenase
jgi.p_Triatrov1_453703	1896	631	transcription factor

The identified *T. atroviride pks1* BGC was used as query to search the publicly available genomes of 25 *Trichoderma* species (supplementary Table S3) with cblaster and clinker (Fig. 4). Orthologous clusters or cluster parts were found in *T. atroviride* IMI206040, *T. gamsii*, and *T. asperellum*, all of which belong to the section *Trichoderma* and are known to produce 6-PP [11, 23, 60, 61], but are absent in other *Trichoderma* species that do not produce 6-PP, such as *T. reesei* and *T. virens*. In *T. gamsii* the entire cluster is present in a conserved synteny but contains an additional uncharacterized gene. In the *T. asperellum* genome, most of the predicted cluster genes are present, though gene order has undergone partial re-arrangements (Fig. 4).

### Expression of genes in the predicted *pks1* cluster

To determine the expression of the cluster genes and the cluster borders, transcript levels of *pks1* and additional cluster genes were comparatively analyzed under two different light conditions using RT-qPCR. As expected, *pks1* was considerably expressed under both light conditions tested with an upregulation upon cultivation of *T. atroviride* under reduced light (RL) conditions compared to growth under light-dark (LD) cycles (Fig. 2). Eight genes were not transcribed at all under light-dark conditions with two of them (ID 489595 and ID 453703) remaining below the detection limit as well under reduced light cultivation, suggesting that they are not part of the BGC. The mRNA levels of two genes (ID 492177 - putative fatty acid desaturase and ID 417170 - protein with a FAD-binding domain) were higher upon fungal growth under light-dark conditions evidencing that they are not co-regulated with *pks1* and hence probably are also not part of the cluster. Among the six genes showing a clear co-regulation with *pks1*, i.e., significant upregulation under reduced light conditions, ID 369211 (encoding a putative phosphorylase) and ID 243072 (a C3H1-zinc finger protein-encoding gene) not only displayed high expression levels similar to *pks1* under reduced light conditions but are also located in close proximity to the Pks1-encoding gene (Fig. 2). Based on transcriptional co-regulation, the obtained data hence evidence that the putative *pks1* BGC consists of six genes, i.e., ID 369211, ID 243072, ID 453700 (*pks1*), ID 243098, ID 314935, and ID 404080.

### Functional and phenotypical characterization of *pks1* by gene deletion

To obtain clarity about the involvement of *pks1* in SM and especially 6-PP biosynthesis, *∆pks1* gene deletion mutants were generated, as deletion of the backbone-forming PKS is expected to result in a complete loss of product formation. To this end, the *pks1* open reading frame was replaced by a hygromycin B resistance cassette by using the CRISPR/Cas9 system. Two gRNAs targeting both the 5’ and the 3’ ends of the *pks1* open reading frame were designed in order to excise the target gene. After three rounds of single spore isolation, three mitotically stable *∆pks1* mutants were obtained and confirmed by PCR-assisted genotyping (supplementary Fig. S1B). The strains were named *∆pks1A*, *∆pks1B*, and *∆pks1C*.

**Fig. 5:**
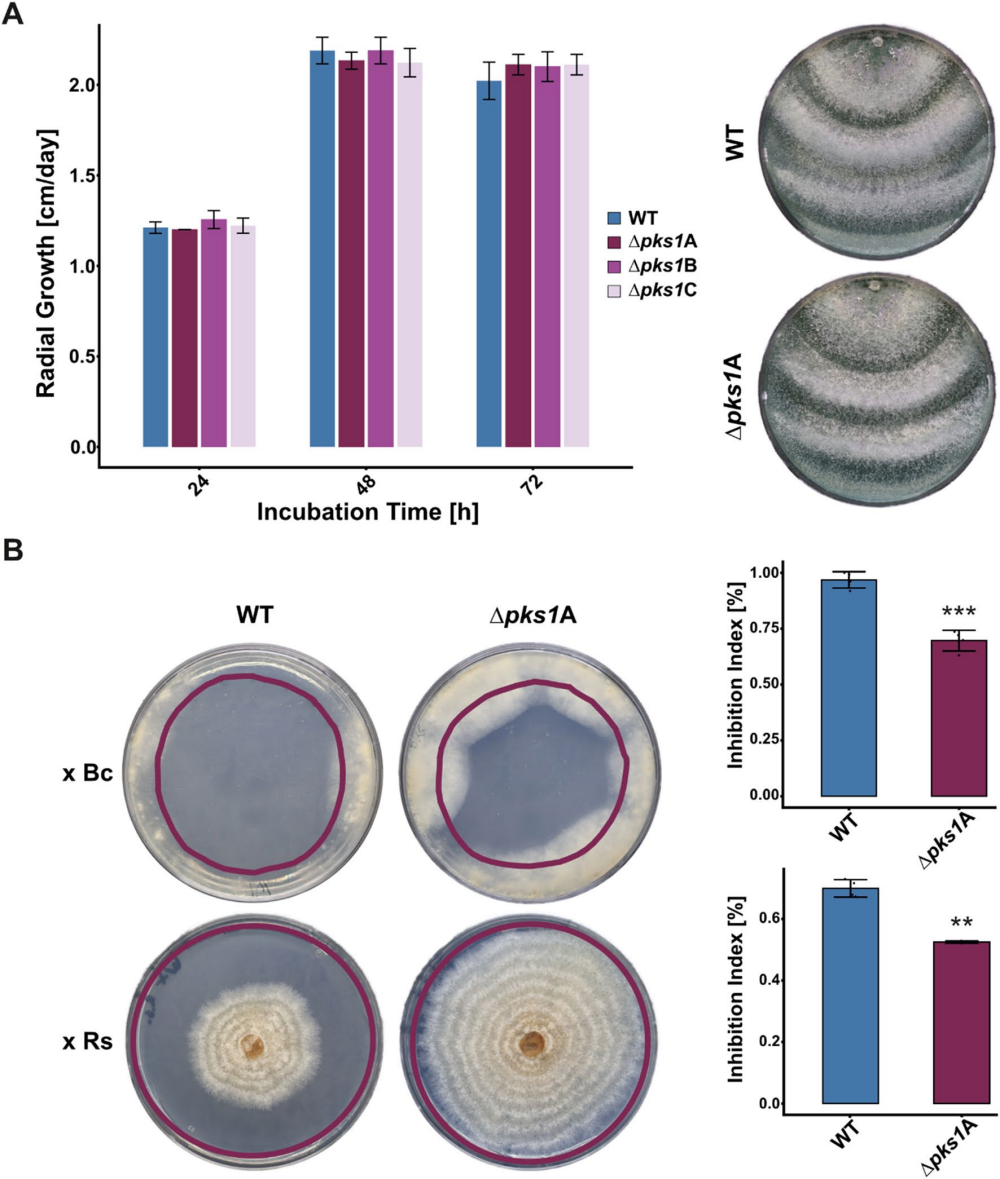
Radial colony growth and antifungal activity of *T. atroviride* wild type in comparison to its ∆*pks1* mutants. (**A**) Colony diameter measurements of *T. atroviride* wild type (WT; blue) and *pks1* deletion mutants *∆pks1*A, *∆pks1*B, *∆pks1*C (purple tones) on PDA at 25 °C under light-dark conditions. No statistically significant differences between the WT and the *∆pks1* mutants were found. A representative image out of three biological replicates (*n* = 3) is shown on the right. (**B**) Inhibition assay to assess the antifungal activity of metabolites secreted by *T. atroviride* WT and *∆pks1*A against *B. cinerea* (Bc; top row) and *R. solani* (Rs; bottom row) on PDA under reduced light conditions. Purple lines indicate the area originally covered by the *T. atroviride* colony after 2 days of growth before the test fungus was inoculated. Representative images out of four biological replicates (*n* = 4) are shown. Inhibition index represents the inhibitory activity of *T. atroviride* WT and *∆pks1*A against the respective fungal test organism. Results shown are means ± standard deviation. The asterisks indicate statistically significant differences of the ∆*pks1*A mutant compared to the WT

Upon cultivation on PDA plates, the three independent *∆pks1* mutants exhibited radial colony extension rates similar to the WT. Furthermore, no alterations in conidiation or other morphological characteristics of the *∆pks1* mutants compared to the WT were observed (Fig. 5A).

As several polyketides including 6-PP exhibit antifungal properties [11, 62–64], the effect of *pks1* gene deletion on the inhibitory activity mediated by secreted substances of *T. atroviride* on the plant pathogens *R. solani* and *B. cinerea* was comparatively assessed in the WT and the *∆pks1* mutant. The antifungal activity of the WT against *B. cinerea* (inhibition index of 0.96 reflecting nearly complete growth inhibition of the test organism) was significantly higher than that of the *∆pks1* mutant (inhibition index of 0.70) (Fig. 5B). Similarly, the inhibition index against *R. solani* was 0.70 for the WT strain, whereas the *∆pks1* mutant showed a reduced inhibition index of 0.50, evidencing a significant contribution of Pks1-derived metabolites to the antifungal activity of *T. atroviride*.

**Fig. 6:**
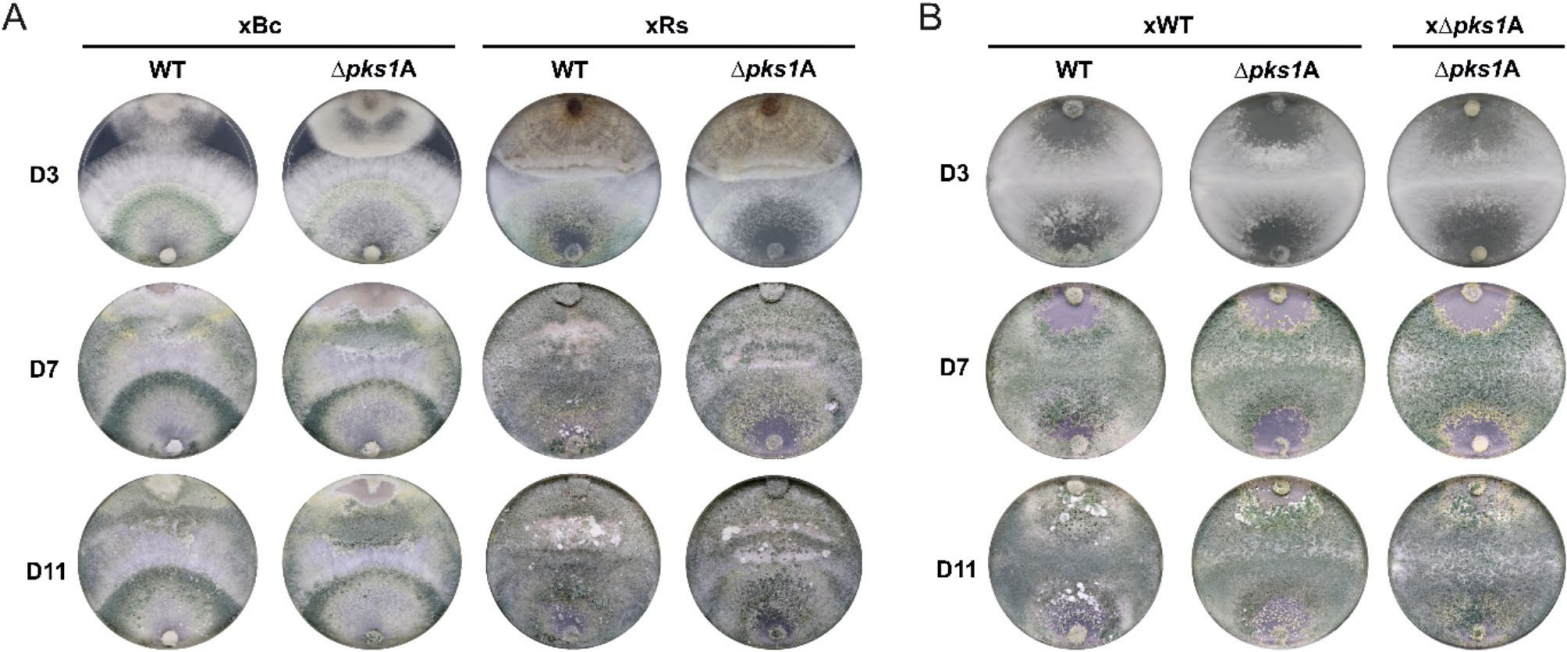
Mycoparasitic interactions and self-confrontations of *T. atroviride* wild type in comparison to its ∆*pks1* mutant. (**A**) The mycoparasitic behavior of *T. atroviride* wild type (WT) and the *∆pks1*A mutant against *B. cinerea* (xBc) and *R. solani* (xRs) was assessed after 3 (D3), 7 (D7), and 11 days (D11) of incubation on PDA under reduced light conditions. On each plate, the *T. atroviride* WT or mutant is inoculated on the bottom and the host fungus on the top. (**B**) Self-confrontations of the WT (xWT) and the *∆pks1* mutant (x*∆pks1*A), as well as confrontations between WT (bottom) and *∆pks1* mutant (top) were assessed after 3 (D3), 7 (D7), and 11 days (D11) of incubation on PDA under reduced light conditions. A representative image out of three biological replicates (*n* = 3) is shown

Based on the observed contribution of *pks1* to the antagonistic activity of *T. atroviride* (Fig. 4), we next comparatively assessed the WT and the *∆pks1* deletion strains in dual culture confrontation assays with either *B. cinerea* or *R. solani* as host fungi (Fig. 6A). While dual culture confrontation assays reflect multiple factors such as resistance, competition, and antagonism, overgrowth is commonly used as an indication of antagonistic activity [65]. Both, the WT and *∆pks1* were able to establish direct contact with the fungal hosts three days post-inoculation. At this stage, *B. cinerea* colony growth appeared to be impaired by the *T. atroviride* WT, but less so by the *∆pks1* mutant. After 7 days, the WT had already completely overgrown *B. cinerea*, while the mutant even after 11 days was not able to fully cover the host colony. In contrast, in confrontations with *R. solani*, both the WT and the *∆pks1* mutants exhibited robust antagonistic activity resulting in a complete overgrowth of the host fungus (Fig. 6A).

Fungal SMs are not only important players in interactions with other organisms but may also act as self-signaling hormones [15, 66]. To assess a putative role of Pks1 and its derived metabolites on the self-interaction of *T. atroviride*, self-confrontations of the WT and *∆pks1* as well as confrontations between the WT and its mutant were performed. While in the WT x WT interaction the two colonies after 11 days had completely grown into each other, subtle deviations were observed in confrontations involving the *∆pks1* mutant. In both WT x *∆pks1* and *∆pks1* x *∆pks1* interactions, separation zones between the interacting colonies with a whitish appearance remained (Fig. 6B), which may indicate differences in mycelial cell fusion potentially caused by the loss of the *pks1* gene.

**Fig. 7:**
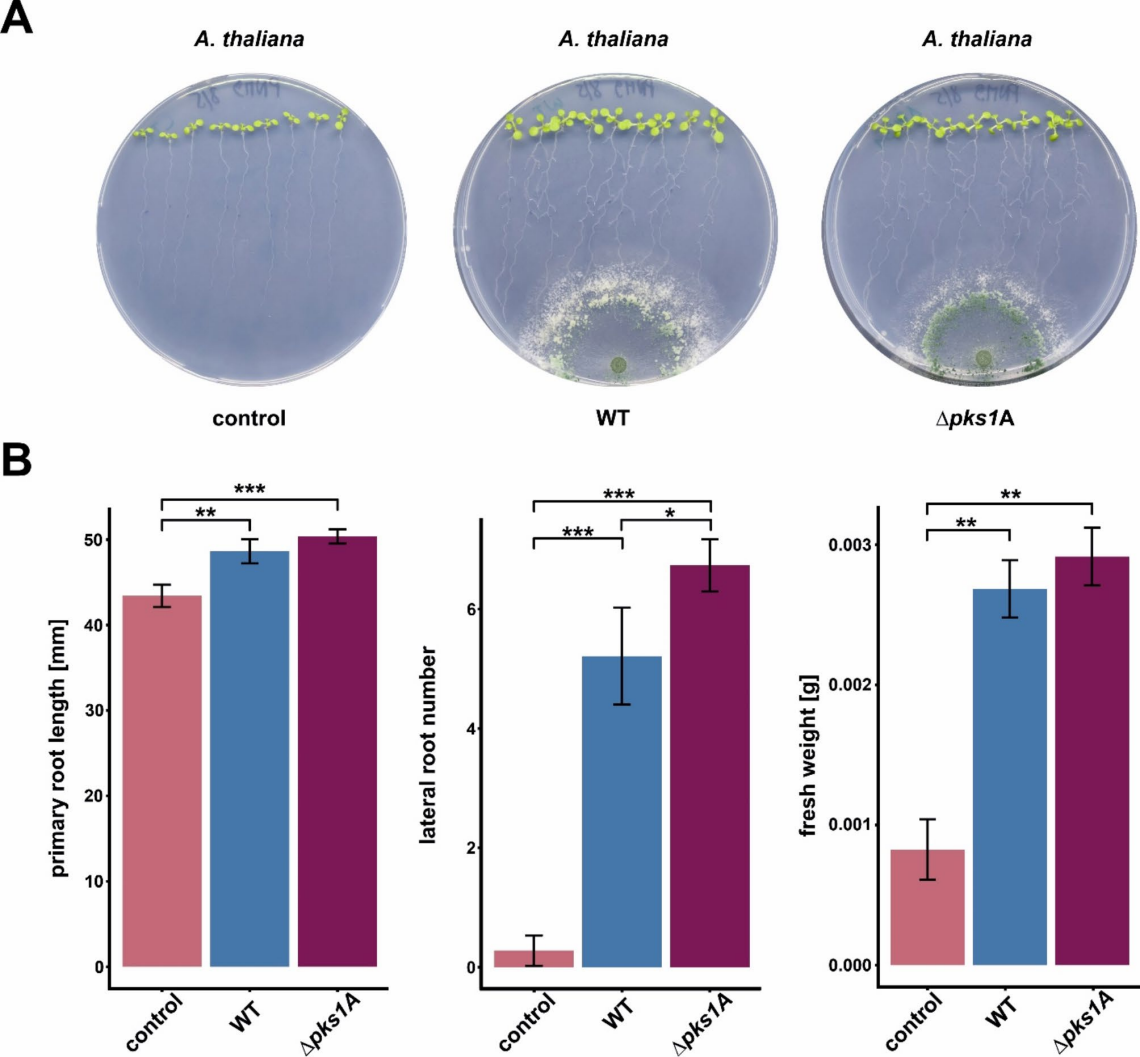
Effects of *pks1* gene deletion in the interaction of *T. atroviride* with *Arabidopsis thaliana.* (**A**) Representative images out of four biological replicates (*n* = 4) showing *A. thaliana* plantlets upon interaction with either the wild type (WT), the *∆pks1* mutant (∆*pks1*A), or without fungal co-cultivation partner (control). Pictures were taken 5 days post-fungal inoculation. (**B**) Quantitative measurements of *A. thaliana* growth parameters. The primary root length (in mm), lateral root number, and fresh weight (in grams) were measured for plants interacting with *T. atroviride* WT or the ∆*pks1*A deletion mutant, and control plants without fungal interaction. Each bar represents the mean ± standard deviation of the mean from four biological replicates. Statistical significance is indicated as follows: *p < 0.05, **p < 0.01, ***p < 0.001

*Trichoderma-*derived SMs have manifold effects on plants including plant growth promotion and activation of plant defense systems [67]. We hence tested the effect of *pks1* gene deletion on the interaction of *T. atroviride* with *A. thaliana* plantlets. When interacting with the WT strain, *A. thaliana* exhibited a substantial increase in primary root length, lateral root number, and fresh weight compared to control plants that were grown alone (Fig. 7). Interestingly, plants interacting with the ∆*pks1* mutant demonstrated even more pronounced improvements in the growth parameters tested. Especially the number of lateral roots developed by the *A. thaliana* plantlets increased significantly upon interaction with the mutant compared to interaction with the WT (Fig. 7B), indicating that *pks1* deletion affects the biosynthesis of plant growth influencing substances and root system architecture, likely due to the absence of 6-PP or other *pks1*-dependent metabolites.

### Targeted metabolomics of the *∆pks1* mutant

**Fig. 8:**
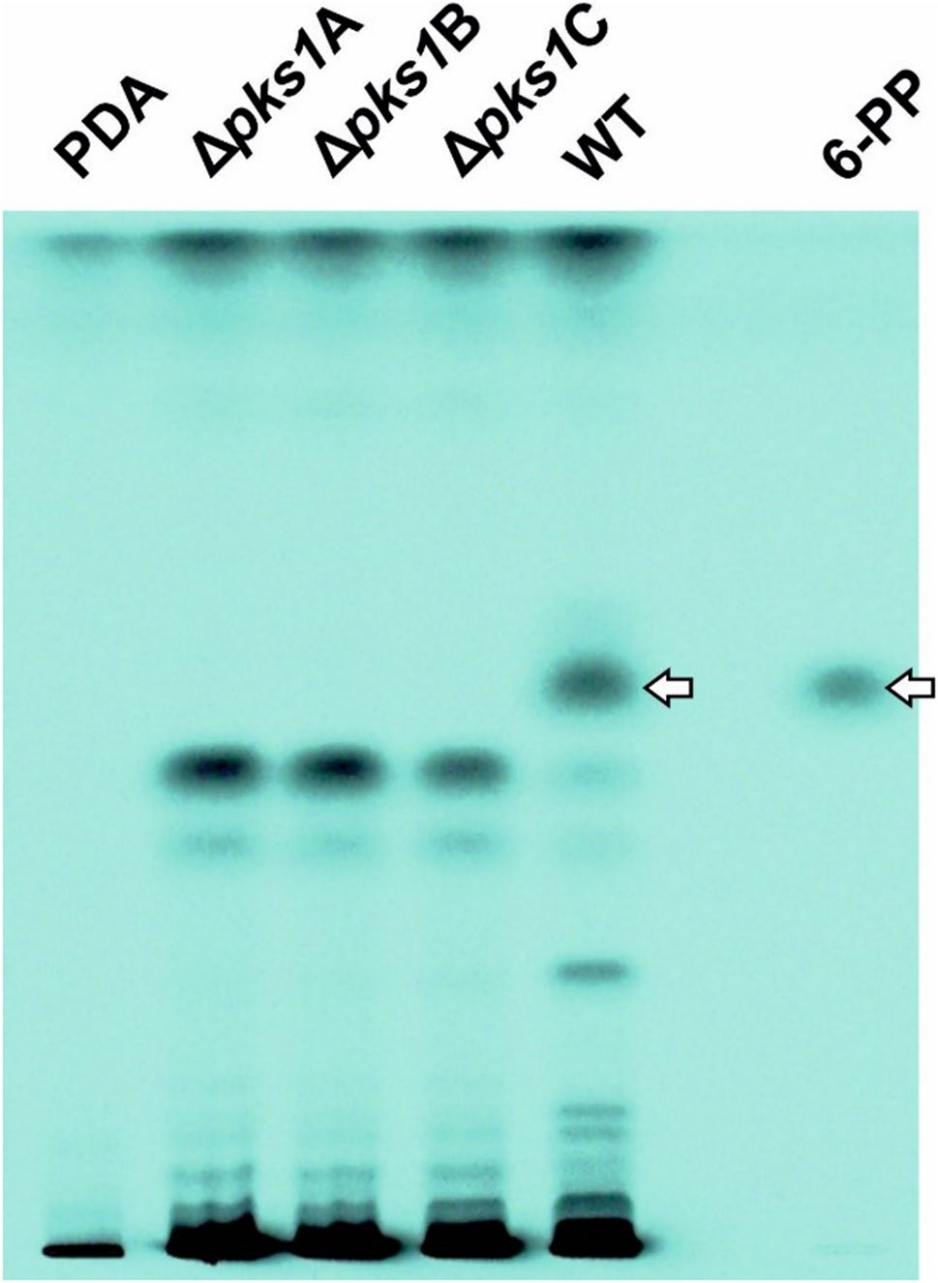
Analysis of 6-pentyl-alpha-pyrone (6-PP) production in *T. atroviride* wild type and ∆*pks1* mutants. High-performance thin-layer chromatography (HPTLC) analysis of extracts from *T. atroviride* WT and *pks1* deletion mutants (∆*pks1*A, ∆*pks1*B, ∆*pks1*C). Control (PDA), WT, and *∆pks1* mutants were incubated for 96 h at 25 °C under reduced light conditions. Metabolites were extracted from the agar and separated by HPTLC. Two µg 6-PP were applied as reference (6-PP). Metabolite patterns were visualized at 254 nm. White arrows indicate the band corresponding to 6-PP in the WT extract and the reference

**Fig. 9:**
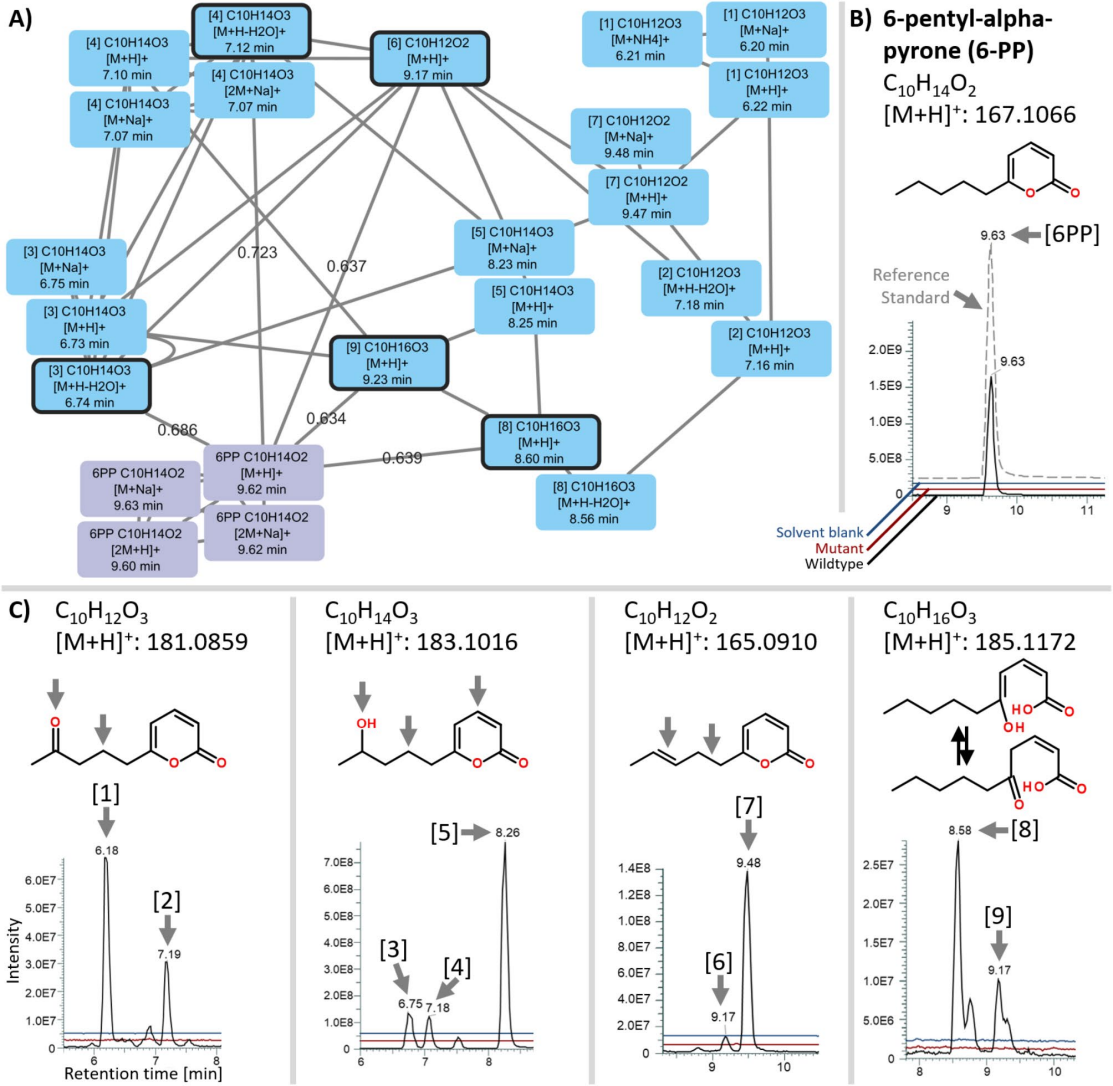
Molecular Network of the detected features of 6-PP and its presumed related biosynthetic products and overlays of extracted ion chromatograms (EICs) corresponding to predicted 6-PP derivatives in *T. atroviride* WT, *pks1* deletion mutant extracts and pure extraction solvent. **A**) Molecular Network of the detected metabolic features of 6-PP and its presumed related biosynthetic products (1) to (9). 6-PP is connected directly with the protonated ion molecules and electrospray-derived in-source fragments of 5 spectral and thus similar structures (scores are shown and metabolic features are highlighted with a black frame). **B**), **C**) Overlays of extracted ion chromatograms (EICs) of 6-PP and compounds (1) to (9) in *T. atroviride* WT, *pks1* deletion mutant extracts and pure extraction solvent as additional control. **C**) from left to right: possible keto-, hydroxy-, dehydrated and hydrolyzed (i.e., with opened lactone ring) 6-PP derivatives. Gray vertical arrows indicate possible sites of chemical modification. The structural relationship of the derivatives with the main product 6-PP is further supported by a large number of MS/MS fragments shared between 6-PP and the illustrated compounds (1) to (9) (Figure S2)

In order to evaluate a putative involvement of Pks1 in the biosynthesis of 6-PP, the metabolite profiles of 96 h old PDA cultures of the *T. atroviride* WT and the three independent *pks1* gene deletion mutants ∆*pks1*A, ∆*pks1*B, and ∆*pks1*C were comparatively analyzed by HPTLC and visualized spectroscopically at a wavelength of 254 nm. In the obtained chromatograms, the band corresponding to 6-PP, as verified by the pure substance as reference on the same plate, could be clearly detected in the WT samples but was completely absent in all three *∆pks1* mutants (Fig. 8). The identity of 6-PP in WT samples and its absence in the ∆*pks1* mutants was further confirmed by targeted LC-HRMS analysis (Fig. 9B).

Moreover, inspection of the HPTLC-chromatograms revealed a number of bands representing additional metabolites being more abundant in the WT extracts compared to those of *∆pks1*. Based on this observation and the fact that partly reduced polyketides often occur as mixtures of closely related chemical structures [68, 69], WT and *∆pks1* extracts were comparatively analyzed by LC-HRMS/MS for the presence of 6-PP derivatives, potentially derived from an altered degree of reduction during iterative formation of the pentaketide 6-PP or its partial oxidation thereafter. Targeted inspection of extracted ion chromatograms (EICs) for the presence of protonated molecules [M + H]^+^ revealed a total of nine putative 6-PP derivatives. The EICs suggested two tentative keto-, three different monohydroxy- and two dehydrated 6-PP derivatives, all of which were present in WT but missing in *∆pks1* extracts. In addition, at least two isomers of 6-PP with hydrolyzed lactone ring were detected in the WT samples (Fig. 9C). Assuming similar ionization efficiencies like 6-PP, the three isomeric monohydroxy 6-PPs clearly dominated among the detected derivatives with abundances between 8 and 50% relative to 6-PP. All other derivatives occurred in smaller proportions. They accounted for only 1–8% of the main product 6-PP. To further confirm the close structural relationship between all candidates and 6-PP, LC-HRMS/MS measurements of predicted [M + H]^+^ ions were carried out. The obtained MS/MS spectra showed a large number of fragments that were shared between authentic 6-PP and its presumed derivatives. Moreover, the product ion spectra of all isomers of the respective putative 6-PP derivatives had numerous fragments in common (supplementary Fig. S2), which further supports the suggested structures.

## Discussion

6-PP production by *Trichoderma* has first been discovered in studies aimed at identification of the major constituent responsible for the characteristic coconut-like odor of certain strains [70]. Subsequent research established a positive correlation between the ability to produce 6-PP and the antagonistic activity of *Trichoderma* fungi against fungal phytopathogens such as *Gaeumanomyces graminis* and *R. solani*, identifying 6-PP as a critical bioactive compound of certain *Trichoderma* species [71, 72].

Initially, it was hypothesized that 6-PP is biosynthesized via a lipoxygenase reaction through beta-oxidation [19], but this theory was later disproved, leaving the metabolic origin of 6-PP elusive [20]. Recent studies suggested that 6-PP biosynthesis involves a polyketide pathway, consistent with a role of PKSs in synthesizing various α-pyrones in other fungi [73].

The phylogenetic analysis presented above suggests that the *T. atroviride* polyketide synthase Pks1 clusters with PKSs known for pyrone formation in other fungi, such as Sol1 of *Alternaria solani* and OtaA of *Aspergillus niger* [55, 74] (Fig. 3). Fungal PKSs synthesizing pyrones typically fall into the class of highly reducing Type I PKSs (HR-Type I PKSs), characterized by the presence of the beta-keto processing domains such as KR, DH, and ER [75]. Accordingly, the identified *T. atroviride* Pks1 enzyme possesses several key catalytic domains, including KR, DH, ER, KS, AT, and methyltransferase (MT), classifying it as HR-Type I PKS (Fig. 4). The AT domain loads the starting unit (e.g. acetate from acetyl-CoA) into the KS domain, repeating the process with extender units (generally malonyl-CoA) to carry out polyketide chain elongation. The KR domain reduces the beta-ketone to the corresponding beta-alcohol, the DH domain subsequently eliminates the beta-alcohol to produce an enoyl thioester, and the ER domain further reduces the enoyl thioester to a saturated acyl thioester. The MT domain introduces methyl groups that contribute to the structural diversity of the polyketide, often methylating the alpha position of the growing polyketide chain before beta-ketoreduction [76, 77]. However, MT activity was not observed in our study, suggesting that the MT domain may be non-functional or a pseudo MT. The absence of a thioesterase (TE) domain in Pks1 suggests an alternative mechanism for the release and cyclization of the polyketide product. The formation of the pyrone ring structure may occur through spontaneous cyclization during hydrolytic cleavage of the polyketide from the PKS, a process observed in systems lacking a TE domain [59]. While the KS domain primarily facilitates chain elongation, it can also catalyze chain release and cyclization [78]. This combination of enzymatic activity and spontaneous chemical processes supports the hypothesis that Pks1 can mediate pyrone ring formation without a TE domain.

Although many *Trichoderma* species are valuable producers of 6-PP, not all possess this capability. For instance, *T. asperellum* IMI393899 and *T. atroviride* strains P1 and IMI206040 have been proven as prolific producers, contributing to their antifungal and growth-promoting effects on plants [11, 23]. In contrast, 6-PP production is absent in *T. reesei* and *T. virens* [9].

In *T. atroviride*, the *pks1* gene is part of an antiSMASH-predicted gene cluster. Using cblaster and clinker to search publicly available genomes of 25 *Trichoderma* species, respective clusters were found in additional species known to produce 6-PP, such as *T. gamsii* and *T. asperellum*, but not in non-producers like *T. reesei* and *T. virens* (Fig. 4). These differences in the ability to biosynthesize 6-PP among different *Trichoderma* species was critical in pinpointing the *pks1* gene as central to 6-PP biosynthesis.

Our previous studies have shown that 6-PP production in *T. atroviride* is highest upon cultivation in darkness [23]. Conversely, exposure to white light, as in light/dark cycles, has an inhibitory effect on 6-PP biosynthesis. Therefore, we investigated the transcript levels of all genes predicted to be part of the *pks1*-containing gene cluster under different light conditions (Fig. 2). The *pks1* gene, along with five additional genes, showed significant upregulation under reduced light compared to cyclic white light conditions, confirming a light-dependent regulatory mechanism of 6-PP biosynthesis. A C3H1-zinc-finger protein-encoding gene (ID 243072) of this cluster was also notably upregulated under reduced light conditions. These proteins are known to bind both DNA and RNA [79], allowing them to regulate gene expression at multiple levels. In plants, C3H1 zinc finger proteins are key regulators in stress responses, growth, and signaling, where they influence the stability, degradation, and translation of mRNA [80, 81]. A similar regulatory mechanism may occur also in *Trichoderma*, where the upregulation of this zinc finger protein under reduced light conditions points to its involvement in fungal adaptation to environmental cues. This aligns with findings in plants and animals, where C3H1-zinc finger proteins respond to phytohormones and other signaling molecules [82]. Interestingly, two other putative transcription factors in the predicted cluster (ID 463177 and 453703) showed no co-regulation with *pks1*, indicating their independence from 6-PP biosynthesis. The predicted *pks1* cluster also includes a lytic polysaccharide monooxygenase (LPMO, ID 314935) encoding gene. LPMOs are involved in the oxidative cleavage of polysaccharides like chitin, cellulose, and hemicellulose in diverse saprophytic as well as plant-interacting microbes [83–85]. The co-localization and co-regulation of the LPMO-encoding gene and *pks1* suggests a coordinated mechanism in *T. atroviride*, where LPMO-mediated degradation of biopolymers is coupled with the antifungal effects of 6-PP. This coordination may not only enhance the ability of *T. atroviride* to parasitize and degrade fungal hosts during mycoparasitism but may also play a critical role in its saprophytic lifestyle by suppressing microbial competitors, allowing more efficient utilization of polysaccharide degradation products through LPMO activity. While genes in closer proximity to the *pks1* core gene showed increased expression under reduced light, genes on both borders of the predicted BGC were only marginally transcribed or their transcription remained under the detection limit. This indicates that these genes are not involved in 6-PP biosynthesis and are not even part of the gene cluster. This is in accordance with the finding that in *T. asperellum* the orthologous genes on the right border of the predicted *T. atroviride* cluster are missing (Fig. 4).

Our results link the *pks1* gene, which is part of a predicted gene cluster, to 6-PP production in *T. atroviride*. 6-PP is considered to be biosynthesized from precursors originating from fatty acid metabolism, e.g. polyunsaturated linoleic acid [19] or hydroxylated ricinoleic acid [86]. It is reasonable to assume that the effect of higher 6-PP production in these fatty acid supplementation experiments was caused by an increased pool of acetyl- and malonyl-CoA resulting from fatty acid degradation, thereby fueling polyketide production. These CoA derivatives are incorporated into the polyketide backbone during the chain elongation process and further modified during each elongation step by the KR, DH and ER domains of the Pks1 enzyme, leading to the final structure of 6-PP. In addition, further specific modifications of the Pks1 product could be catalyzed by enzymes encoded by other genes within the putative *pks1* gene cluster. The predicted dehydrogenase might introduce double bonds, which could subsequently undergo hydration by the presumed hydratase and oxidation by the predicted alcohol dehydrogenase to form monohydroxy derivatives. This aligns with the biosynthetic flexibility seen in HR-Type I PKSs, where tailoring enzymes diversify core structures [27]. Variations of 6-PP may also derive from incomplete reduction of enzyme-bound intermediates by the KR, DH, and ER domains of Pks1, resulting in additional double bonds or hydroxyl groups [87]. During the synthesis of highly reduced polyketides like 6-PP, it can happen that some of the enzymatic steps between C2-unit chain elongations (i.e., KR, DH, ER) are omitted. In such cases minor fractions of the polyketides finally released from the PKS will consist of incompletely reduced derivatives with keto- or hydroxyl groups or double bonds. Comparative targeted extraction of predicted LC-HRMS/MS ion chromatograms (EICs) revealed several candidates which were present in WT but not in ∆*pks1* mutant culture filtrates (Fig. 9C). The illustrated EICs and LC-HRMS/MS fragment patterns were in agreement with 6-PP derivatives exhibiting an additional keto- or hydroxyl group or an extra double bond in their chemical structure. Moreover, enzymatic modifications such as lactone ring opening or hydroxylation could involve enzymes outside the *pks1* cluster, or occur via non-enzymatic reactions driven by chemical instability or environmental factors.

∆*pks1* mutants showed radial growth rates and morphological characteristics similar to the WT, supporting the common assumption that SMs primarily enhance ecological fitness rather than impacting growth or reproduction (Fig. 5A) [62]. This is in accordance with the previously described activities of 6-PP as antifungal, antibacterial, and signaling molecule, enhancing *Trichoderma’s* ecological competitiveness and contributing to its biocontrol activity [13, 88]. Here, we demonstrated that loss of *pks1* results in a significant reduction of the inhibitory activity against *B. cinerea* and *R. solani* (Fig. 5B), indicating that Pks1-derived metabolites substantially contribute to the antifungal activity mediated by a cocktail of substances secreted by *T. atroviride*. A role of Pks1 in antagonism is further supported by an earlier study that has found *pks1* among the genes that are upregulated during the mycoparasitic interaction of *T. atroviride* IMI206040 with *R. solani* [89]. Moreover, 6-PP levels increased in the presence of *B. cinerea* or *Fusarium culmorum* [90, 91]. Our confrontation assays indicated that while both, the *T. atroviride* WT and ∆*pks1* strains, could establish direct contact with the phytopathogens *B. cinerea* and *R. solani*, the WT strain displayed superior mycoparasitic activity (Fig. 6A). Specifically, the WT inhibited *B. cinerea* already in the pre-contact stage and then completely overgrew the plant pathogen within a few days, whereas the ∆*pks1* mutant was unable to fully cover the host colony. In confrontations with *R. solani*, both strains exhibited robust mycoparasitic activity, suggesting that Pks1-derived metabolites are more critical for antagonism against *B. cinerea* than *R. solani*.

The subtle deviations between the WT and its ∆*pks1* mutant observed in *Trichoderma-Trichoderma* self-confrontation assays (Fig. 6B) indicate a role of Pks1-derived substances in self-signaling and mycelial cell fusion. In particular, the formation of demarcation zones with a whitish appearance in self-interactions involving the ∆*pks1* mutant evidences that the loss of the *pks1* gene may affect vegetative compatibility and cell fusion processes within *T. atroviride* [92]. This finding aligns well with a study indicating involvement of 6-PP in self-signaling and hypothesizing that the morphological changes induced by 6-PP are required to launch a localized mycoparasitic attack [15]. However, further studies employing microscopic techniques will be necessary to conclusively validate these observations.

In addition to the direct interactions with plant pathogens, some *Trichoderma* species are also able to colonize root surfaces and cause substantial changes in plant metabolism, thus promoting plant growth, increasing nutrient uptake and improving crop production [93]. SMs from various *Trichoderma* species, including 6-PP, can increase biomass, height, and chlorophyll content in plants like *A. thaliana* [94, 95]. 6-PP has been reported to show phytohormone (i.e., auxin)-like growth promotion activity in a concentration-dependent manner [13, 96]. Recently, it has been shown that *A. thaliana* seedlings induce the biosynthesis of 6-PP in *T. atroviride* IMI206040, thereby suggesting that plant-derived signals can stimulate metabolite production in the fungus [97]. Given the role of C3H1 zinc finger proteins in plants, the C3H1 zinc finger protein encoded within the *pks1* gene cluster may respond to phytohormones or other signaling molecules secreted by *A. thaliana*, regulating the expression of genes involved in 6-PP biosynthesis. Additionally, the presence of an LPMO-encoding gene within the cluster that is co-regulated with *pks1* in a light-dependent manner points to simultaneously enhanced 6-PP production and polysaccharide degradation. The latter is known to be influenced by light, phytohormones, and other plant exudates [83]. Notably, the AA9 family LPMO plays a key role in plant-fungal interactions by generating cellulose-oxidized oligosaccharides, which trigger immunity responses in plants such as *A. thaliana* [98]. This points to a potential cross-kingdom communication mechanism, where plants not only influence fungal behavior but may actively enhance the production of beneficial metabolites, improving their own growth and resilience [82]. Interestingly, co-culturing *A. thaliana* with the *T. atroviride* P1 ∆*pks1* mutant significantly increased lateral root formation, contrary to previous studies indicating that 6-PP induces lateral root formation and inhibits primary root growth (Fig. 7) [97]. This suggests that the absence of 6-PP or other *pks1*-derived metabolites removes a growth-inhibitory effect, or that *pks1* deletion alters fungal metabolism in a way that promotes root branching. The discrepancy may also be due to plant age and 6-PP concentration, factors influencing the effect of *Trichoderma* on plants [99, 100].

## Conclusions

Our study revealed the polyketide nature of the highly bioactive metabolite 6-PP and led to the identification of the polyketide synthase and its associated gene cluster responsible for its biosynthesis in the mycoparasite *Trichoderma atroviride*. The *pks1* gene turned out to be pivotal for 6-PP production and the absence of this compound in a respective ∆*pks1* mutant correlated with diminished antifungal activity against the plant pathogens *Botrytis cinerea* and *Rhizoctonia solani*, underscoring the importance of Pks1-derived metabolites in biocontrol strategies. Interestingly, the ∆*pks1* mutant induced increased lateral root formation in *Arabidopsis thaliana*, suggesting a more complex interplay of fungal metabolites and plant responses, beyond 6-PP. Functional characterization of other genes within the *pks1* cluster, particularly those encoding regulatory proteins such as the identified C3H1-type zinc finger protein and the LPMO, will be crucial for understanding the molecular mechanisms governing the biosynthesis of 6-PP.

## Electronic supplementary material

Below is the link to the electronic supplementary material.

Supplementary Material 1: Supplementary Table S1: Primers used for transformation and genotyping. Supplementary Table S2: Primers used for qPCR. Supplementary Table S3: *Trichoderma* genomes used for cluster comparison. Supplementary Table S4: Details of statistical analysis given in the figures.)

Supplementary Material 2: Supplementary Figure S1: Gene deletion strategy and verification of *pks1* gene deletion in *Trichoderma atroviride* P1. (A) Locus-specific integration of the split-marker deletion cassette consisting of HY and YG fragments. Orange arrows on top represent CRISPR cutting sites. (B) The absence of the *pks1* gene in the deletion mutants was confirmed using two *pks1* locus-specific primer pairs: 453700_locus-5_F + 453700_locus-5_R (gl1; 3557 bp) and 453700_locus-3_F + 453700_locus-3_R (gl2; 2668 bp). Conversely, the presence of the split-marker deletion cassette was verified with two cassette-specific primer pairs: 453700_locus-5_F + Pgapdh-hph-R (hl1; 1397 bp) and Pgapdh-hph-F + 453700_locus-3_R (hl2; 3568 bp). Genotyping gels were analysed for the three independent* pks1* deletion mutants (Δpks1A, Δpks1B, Δpks1C) and the wild-type control (WT).

Supplementary Material 3: Supplementary Figure S2

## Data Availability

Data is provided within the manuscript or supplementary information files.
