# Supplementary material for: Linking a polyketide synthase gene cluster to 6-pentyl-alpha-pyrone, a Trichoderma metabolite with diverse bioactivities
Source: Microb Cell Fact. 2025 Apr 21;24:89. doi: 10.1186/s12934-025-02718-9 (PMC12010586; doi:10.1186/s12934-025-02718-9)
Supplement: Supplementary file 2 — Supplementary Material 2: Supplementary Figure S1: Gene deletion strategy and verification of pks1 gene deletion in Trichoderma atroviride P1. (A) Locus-specific integration of the split-marker deletion cassette consisting of HY and YG fragments. Orange arrows on top represent CRISPR cutting sites. (B) The absence of the pks1 gene in the deletion mutants was confirmed using two pks1 locus-specific primer pairs: 453700_locus-5_F + 453700_locus-5_R (gl1; 3557 bp) and 453700_locus-3_F + 453700_locus-3_R (gl2; 2668 bp). Conversely, the presence of the split-marker deletion cassette was verified with two cassette-specific primer pairs: 453700_locus-5_F + Pgapdh-hph-R (hl1; 1397 bp) and Pgapdh-hph-F + 453700_locus-3_R (hl2; 3568 bp). Genotyping gels were analysed for the three independent pks1 deletion mutants (Δpks1A, Δpks1B, Δpks1C) and the wild-type control (WT). [file 12934_2025_2718_MOESM2_ESM.pdf]

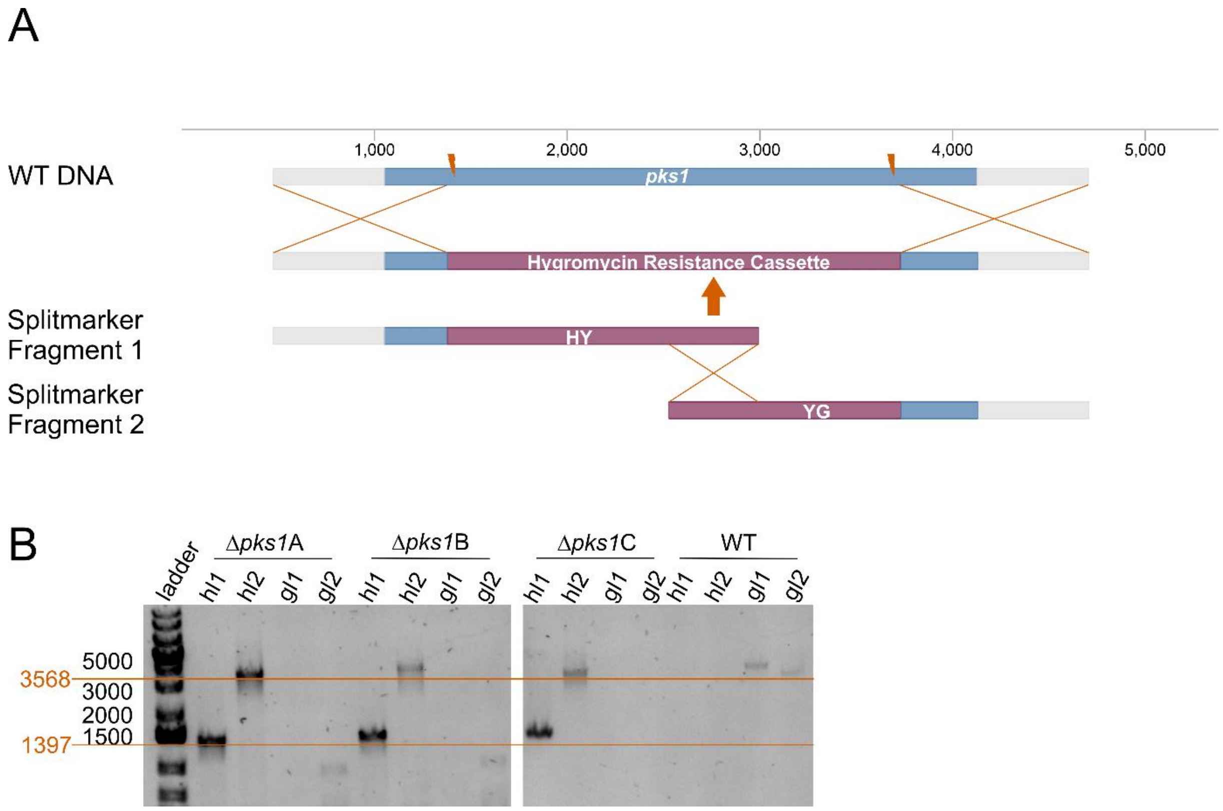

Fig. S1: Gene deletion strategy and verification of *pks1* gene deletion in *Trichoderma atroviride* P1. (A) Locus-specific integration of the split-marker deletion cassette consisting of HY and YG fragments. Orange arrows on top represent CRISPR cutting sites. (B) The absence of the *pks1* gene in the deletion mutants was confirmed using two *pks1* locus-specific primer pairs: 453700_locus-5_F + 453700_locus-5_R (gl1; 3557 bp) and 453700_locus-3_F + 453700_locus-3_R (gl2; 2668 bp). Conversely, the presence of the split-marker deletion cassette was verified with two cassette-specific primer pairs: 453700_locus-5_F + Pgapdh-hph-R (hl1; 1397 bp) and Pgapdh-hph-F + 453700_locus-3_R (hl2; 3568 bp). Genotyping gels were analysed for the three independent *pks1* deletion mutants (Δ*pks1*A, Δ*pks1*B, Δ*pks1*C) and the wild-type control (WT).
