# Supplementary material for: Linking a polyketide synthase gene cluster to 6-pentyl-alpha-pyrone, a Trichoderma metabolite with diverse bioactivities
Source: Microb Cell Fact. 2025 Apr 21;24:89. doi: 10.1186/s12934-025-02718-9 (PMC12010586; doi:10.1186/s12934-025-02718-9)

MS/MS fragment spectrum
6-PP reference standard

[M+H]$^+$, *m/z* 167.1066
RT 9.63min

6-PP

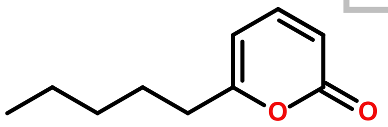

$C_{10}H_{14}O_2$

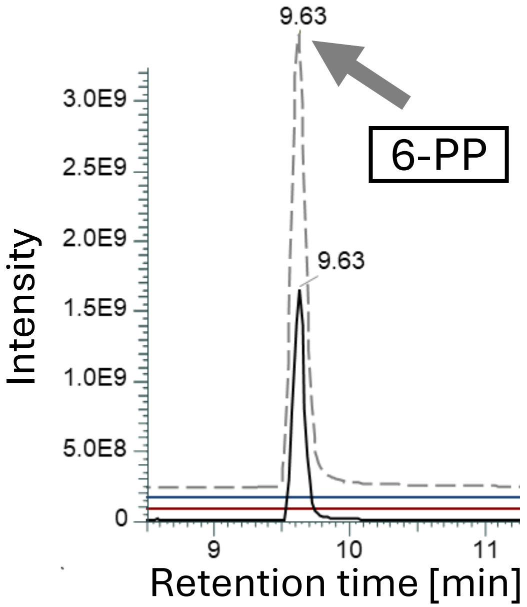

6PP_Standard_MSMS #5996 RT: 9.63 AV: 1 NL: 1.47E9
T: FTMS + p ESI d Full ms2 167.2183@hcd45.00 [50.0000-190.0000]

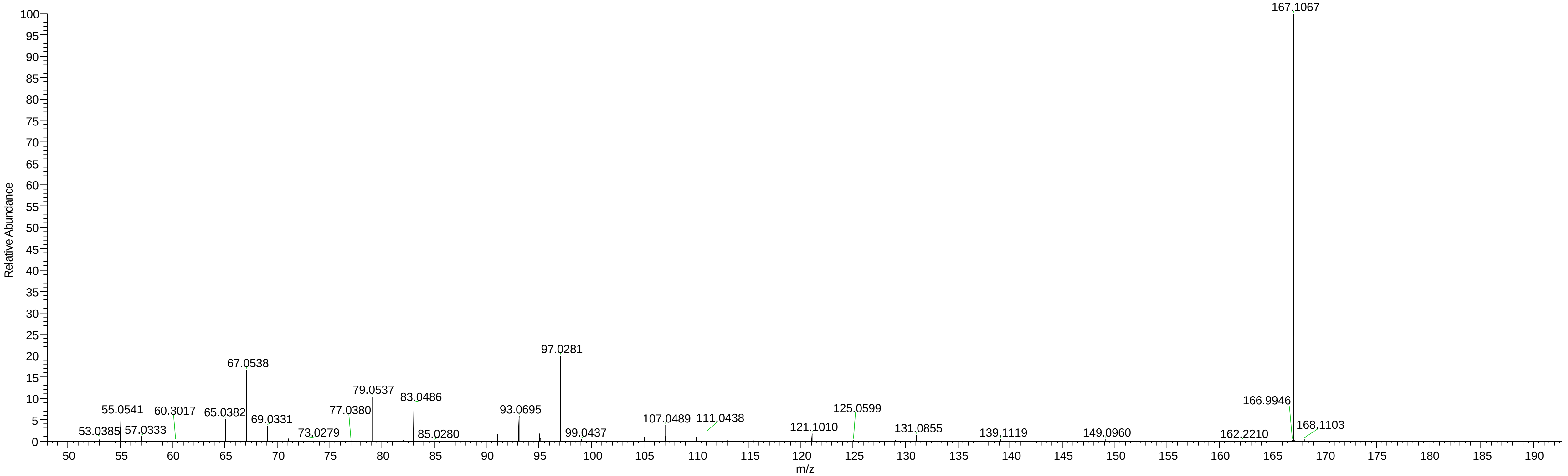

MS/MS fragment spectrum
Wild Type sample

6-PP

[M+H]$^+$, *m/z* 167.1066
RT 9.63min

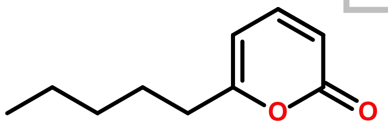

$C_{10}H_{14}O_2$

Fragments present in 6-PP reference standard

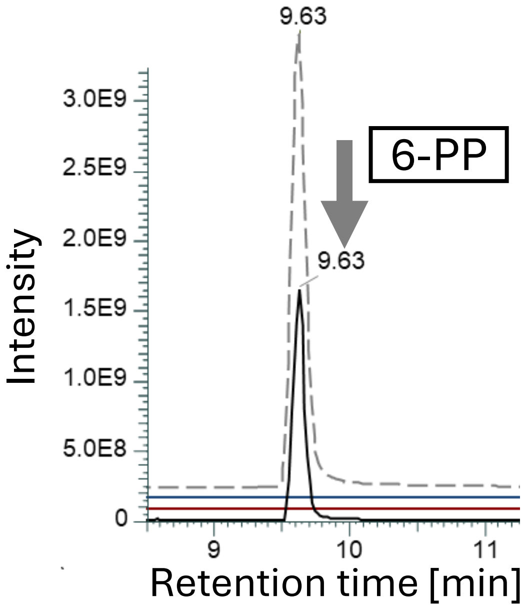

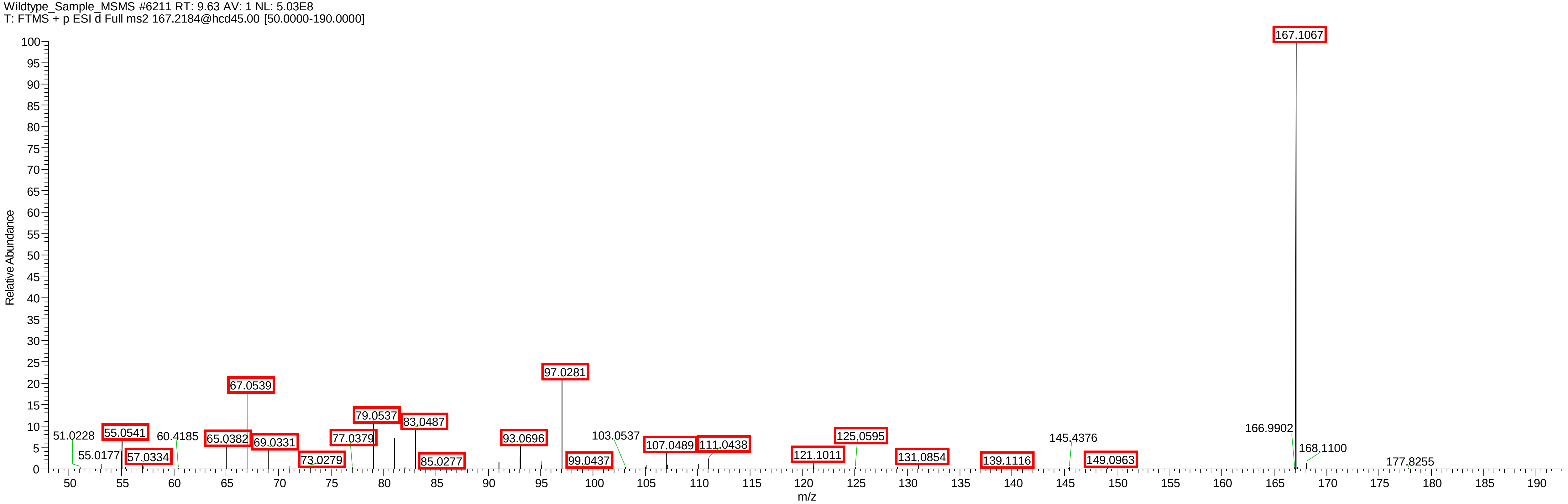

MS/MS fragment spectrum
Wild Type sample

[M+H]$^+$, *m/z* 181.0859
RT 6.18min

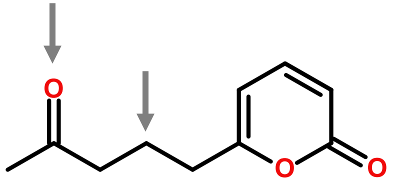

$C_{10}H_{12}O_3$

1

Fragments shared between all isomers of this putative structure

Fragments present in 6-PP reference standard

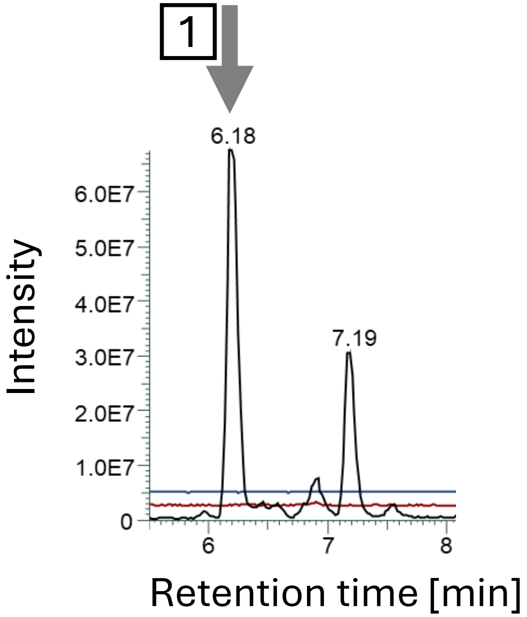

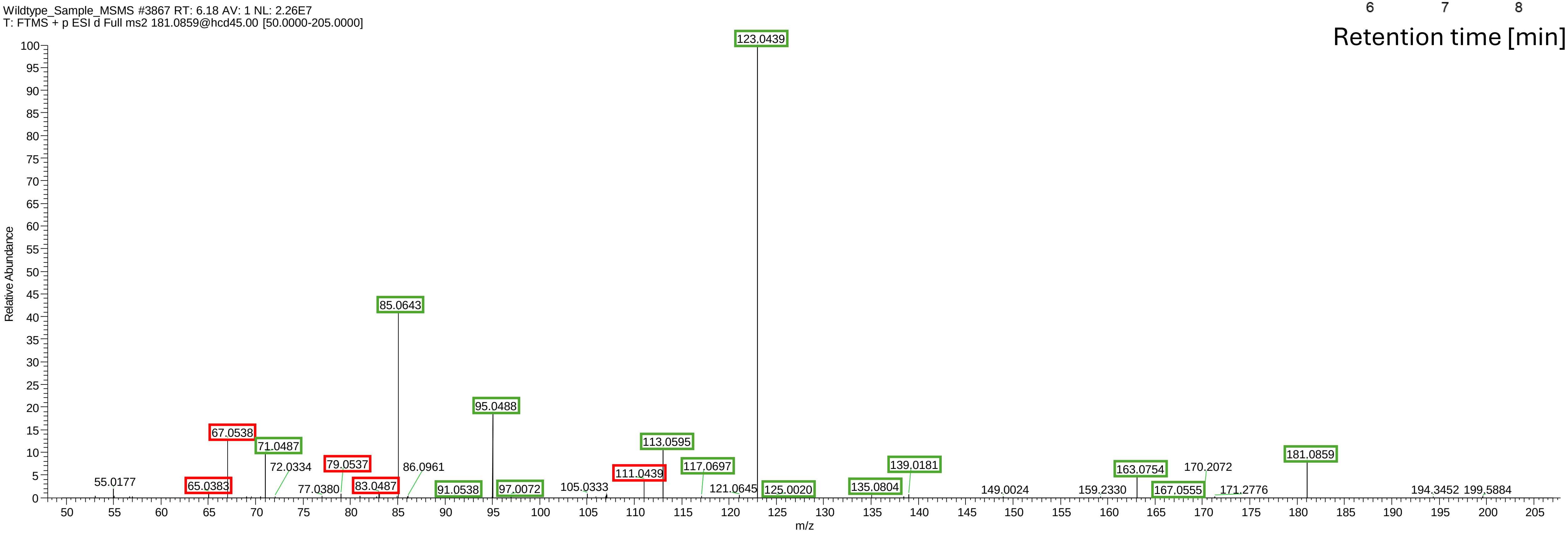

MS/MS fragment spectrum
Wild Type sample

2

[M+H]$^+$, *m/z* 181.0859
RT 7.16min

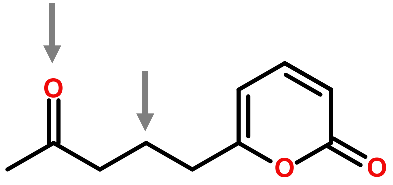

$C_{10}H_{12}O_3$

Fragments shared between all isomers of this putative structure

Fragments present in 6-PP reference standard

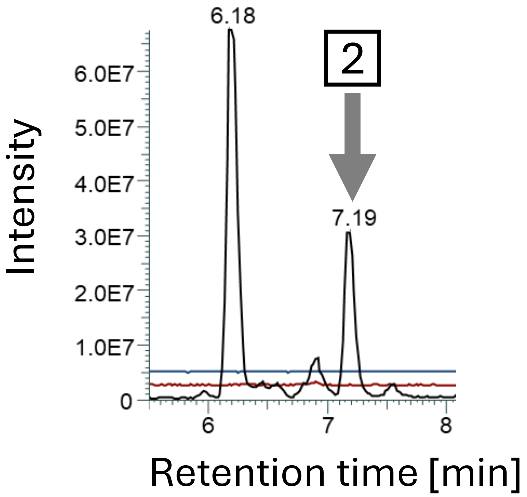

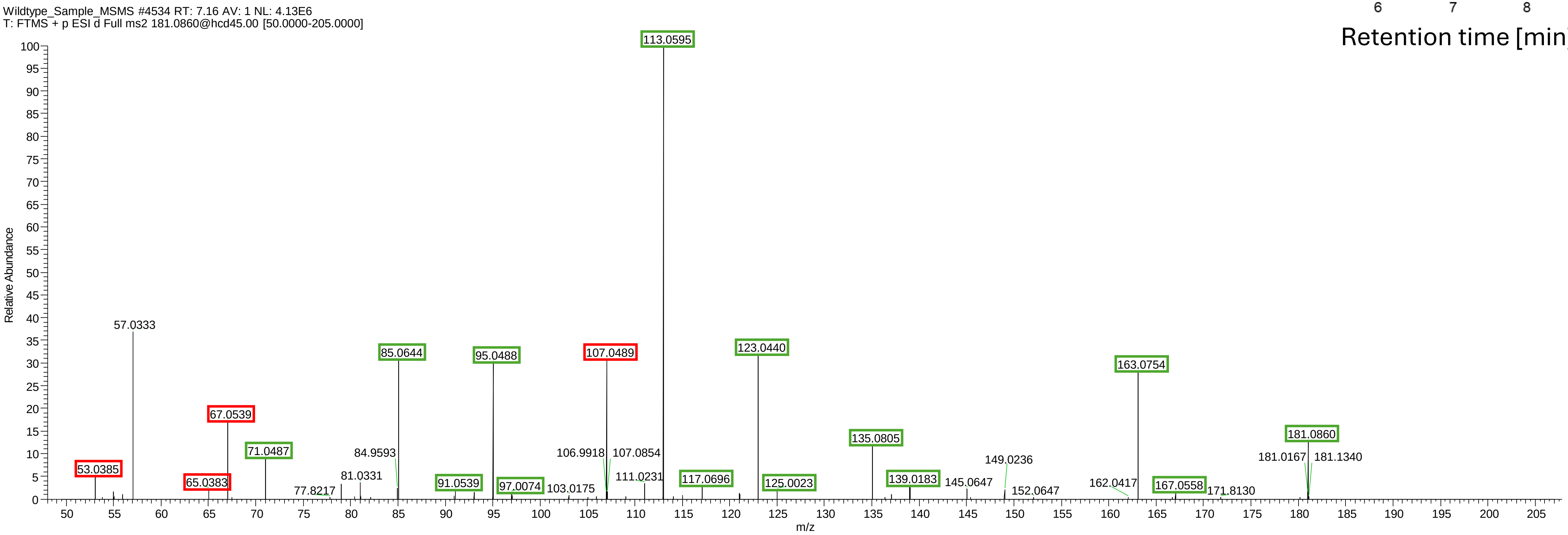

MS/MS fragment spectrum
Wild Type sample

3

$[M+H]^+$, *m/z* 183.1016
RT 6.78min

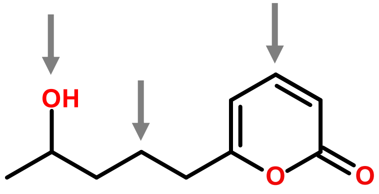

$C_{10}H_{14}O_3$

Fragments shared between all isomers of this putative structure

Fragments present in 6-PP reference standard

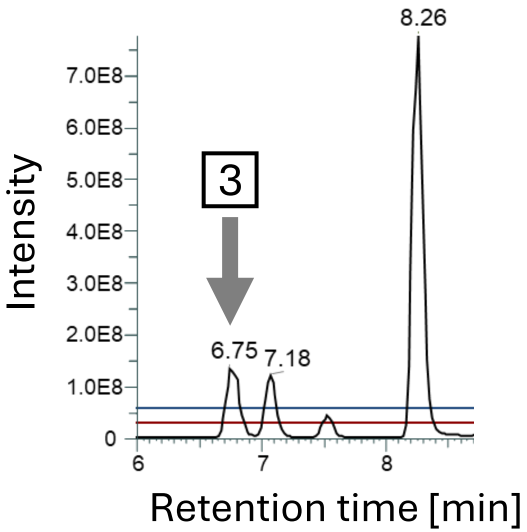

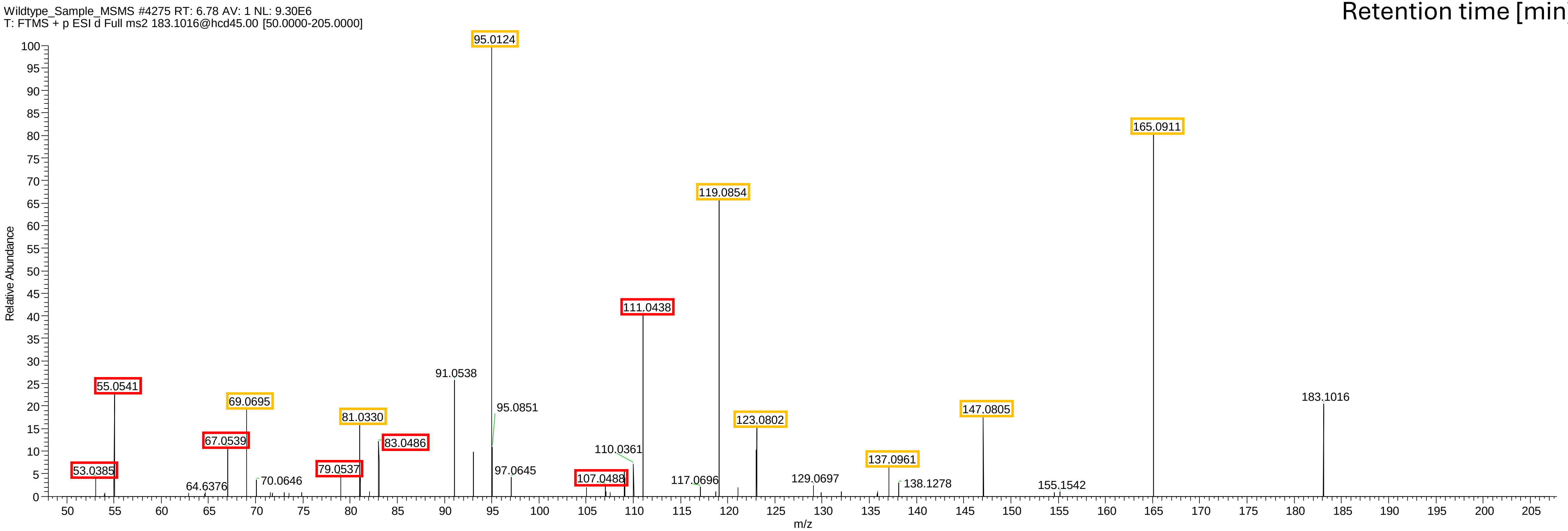

MS/MS fragment spectrum
Wild Type sample

$[M+H]^+$, *m/z* 183.1016
RT 7.07min

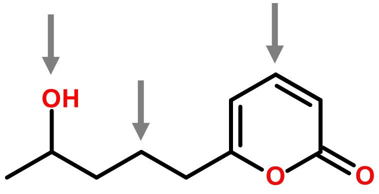

$C_{10}H_{14}O_3$

4

Fragments shared between all isomers of this putative structure

Fragments present in 6-PP reference standard

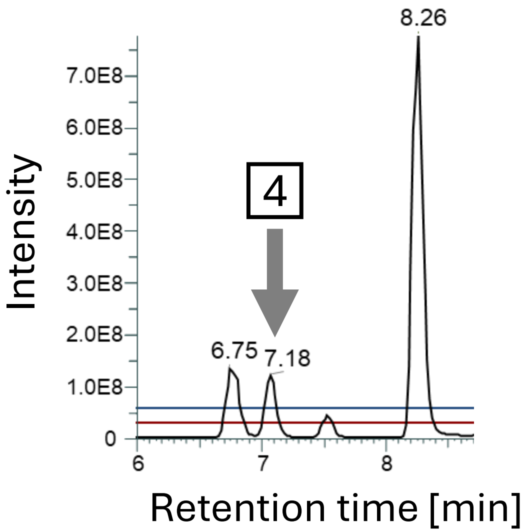

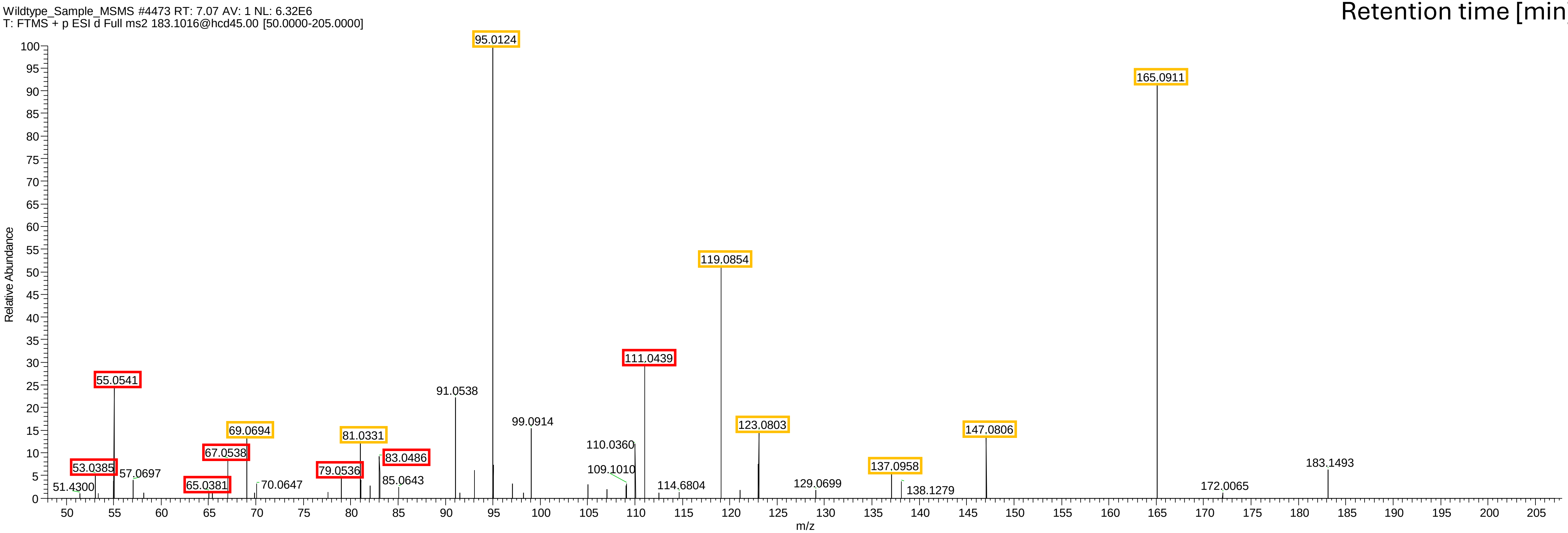

MS/MS fragment spectrum
Wild Type sample

5

[M+H]$^+$, *m/z* 183.1016
RT 8.26min

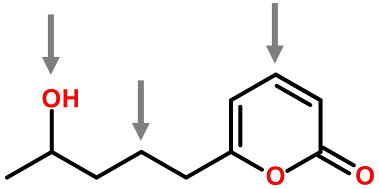

$C_{10}H_{14}O_3$

Fragments shared between all isomers of this putative structure

Fragments present in 6-PP reference standard

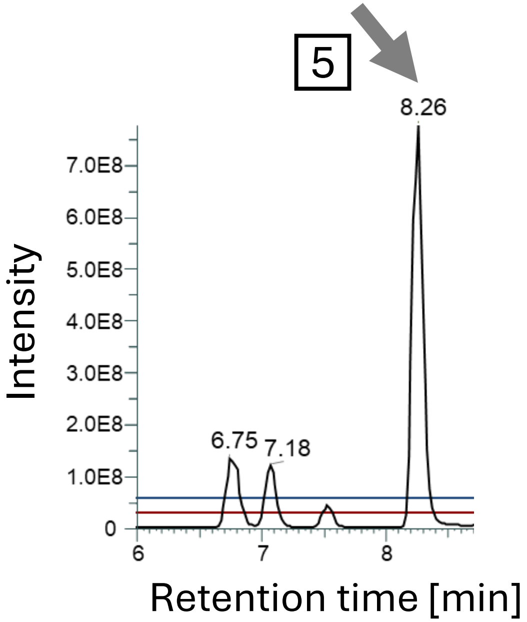

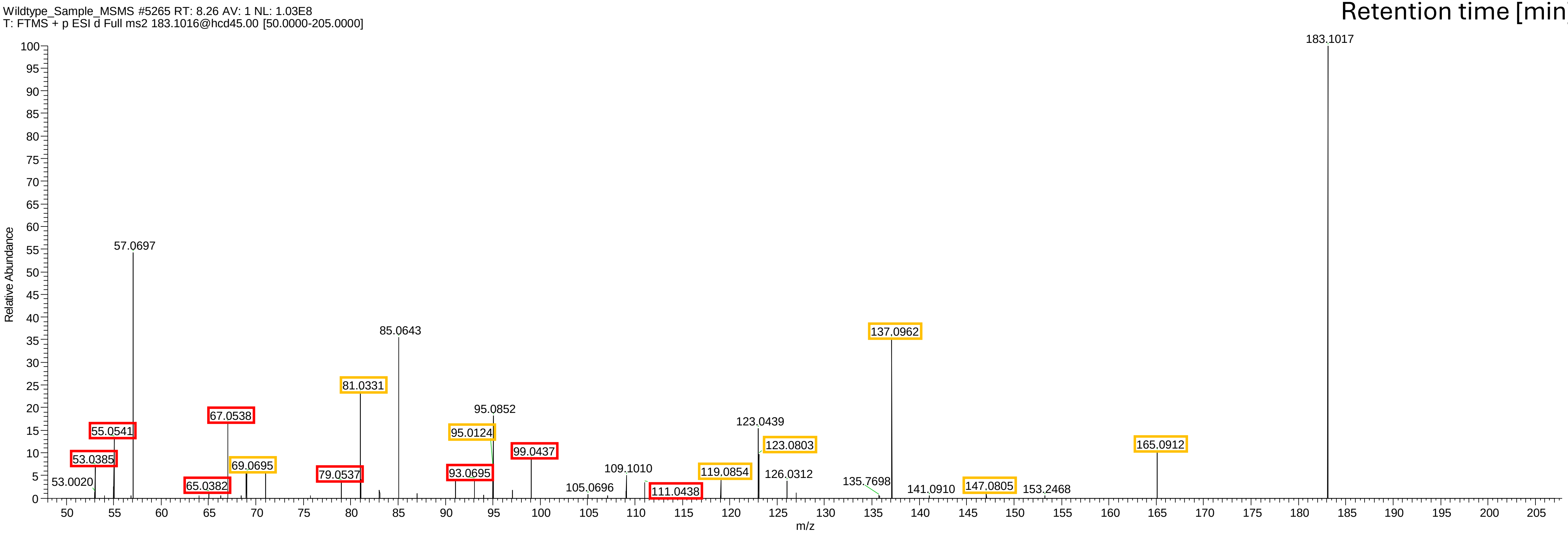

MS/MS fragment spectrum
Wild Type sample

6

$[M+H]^+$, *m/z* 165.0910
RT 9.17min

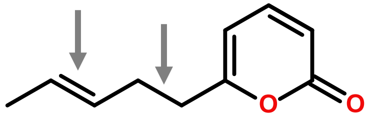

$C_{10}H_{12}O_2$

Fragments shared between all isomers of this putative structure

Fragments present in 6-PP reference standard

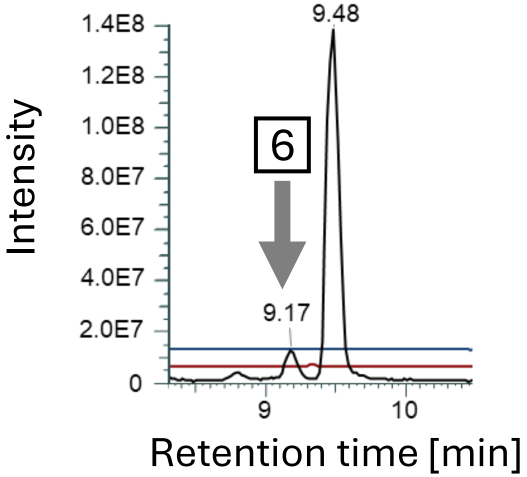

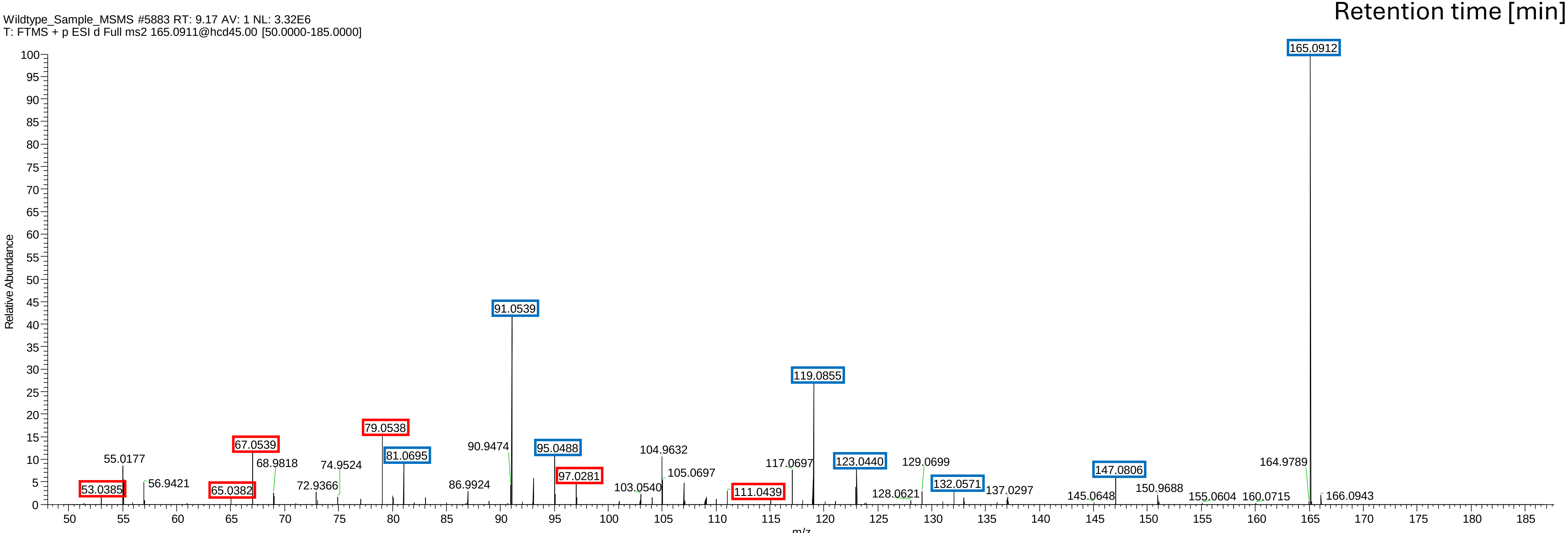

MS/MS fragment spectrum
Wild Type sample

7

$[M+H]^+$, *m/z* 165.0910
RT 9.49min

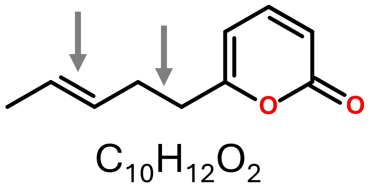

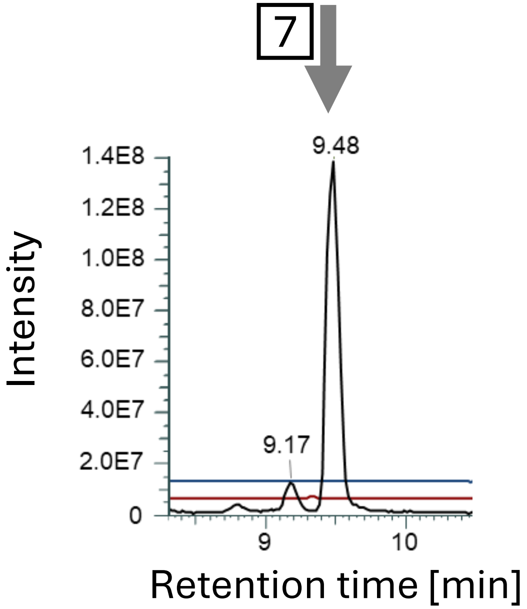

Fragments shared between all isomers of this putative structure

Fragments present in 6-PP reference standard

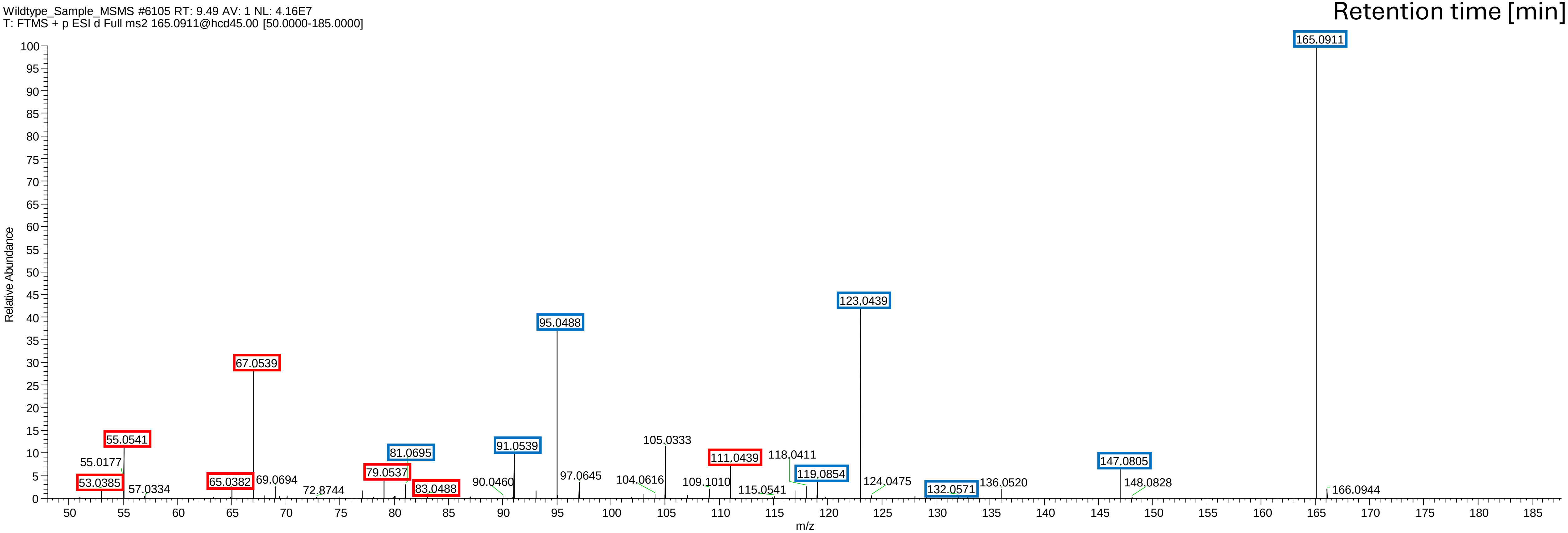

MS/MS fragment spectrum
Wild Type sample

[M+H]$^+$, *m/z* 185.1172
RT 8.56min

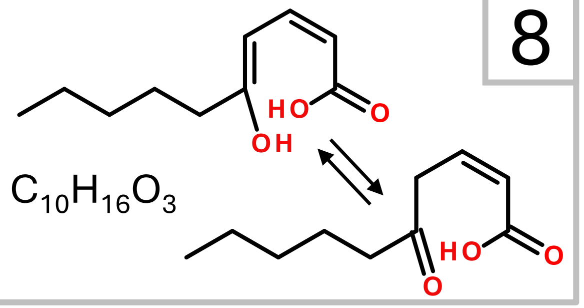

8

Fragments shared between all isomers of this putative structure

Fragments present in 6-PP reference standard

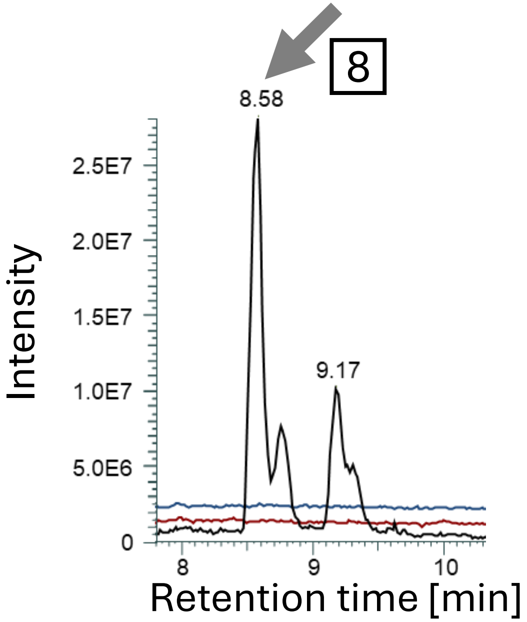

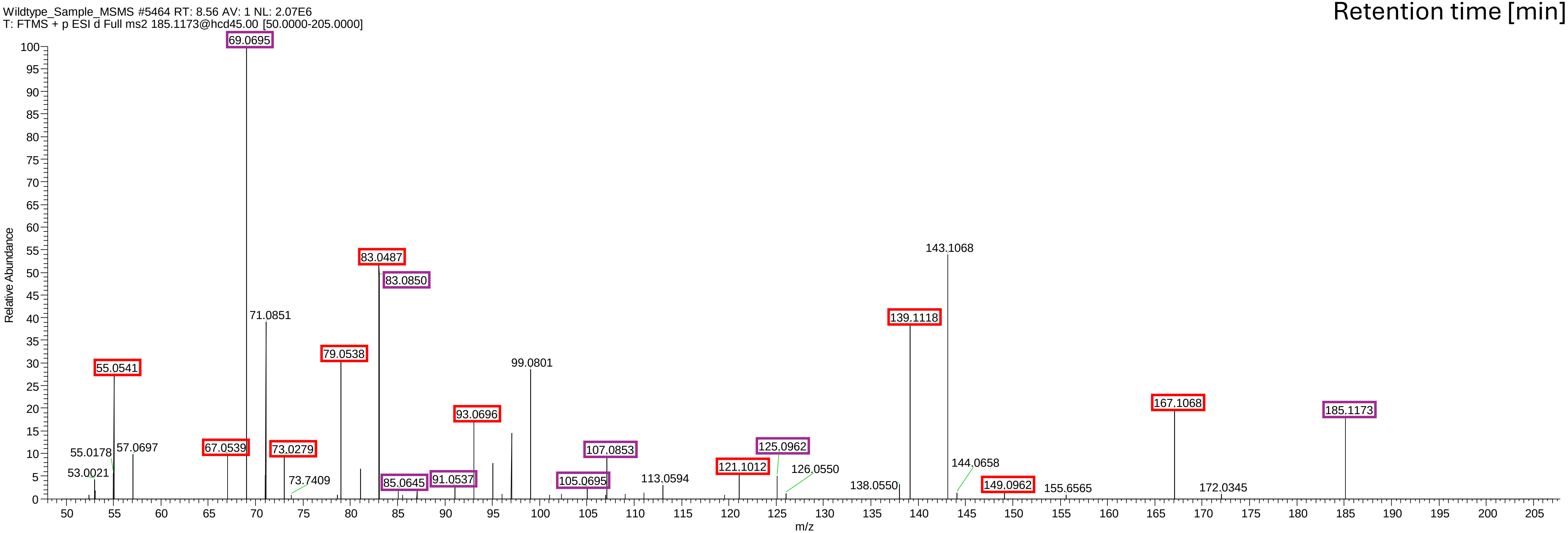

MS/MS fragment spectrum
Wild Type sample

[M+H]$^+$, *m/z* 185.1172
RT 9.17min

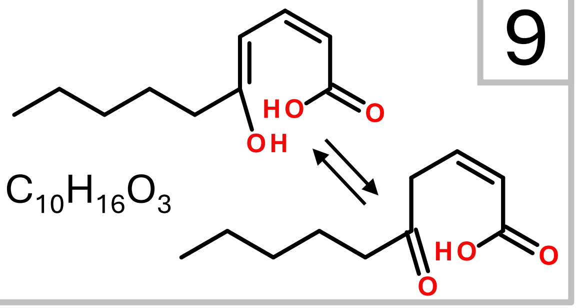

$C_{10}H_{16}O_3$

9

Fragments shared between all isomers of this putative structure

Fragments present in 6-PP reference standard

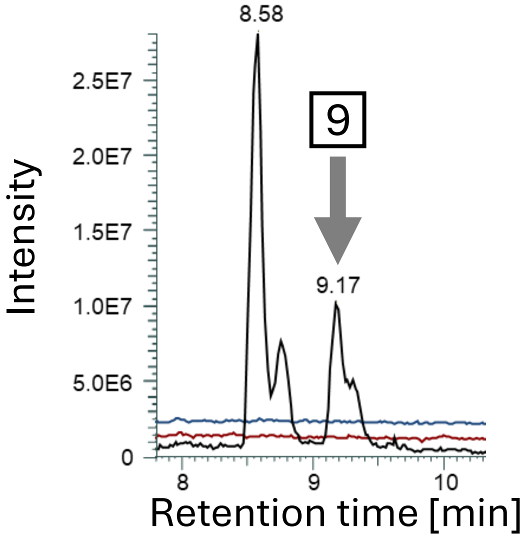

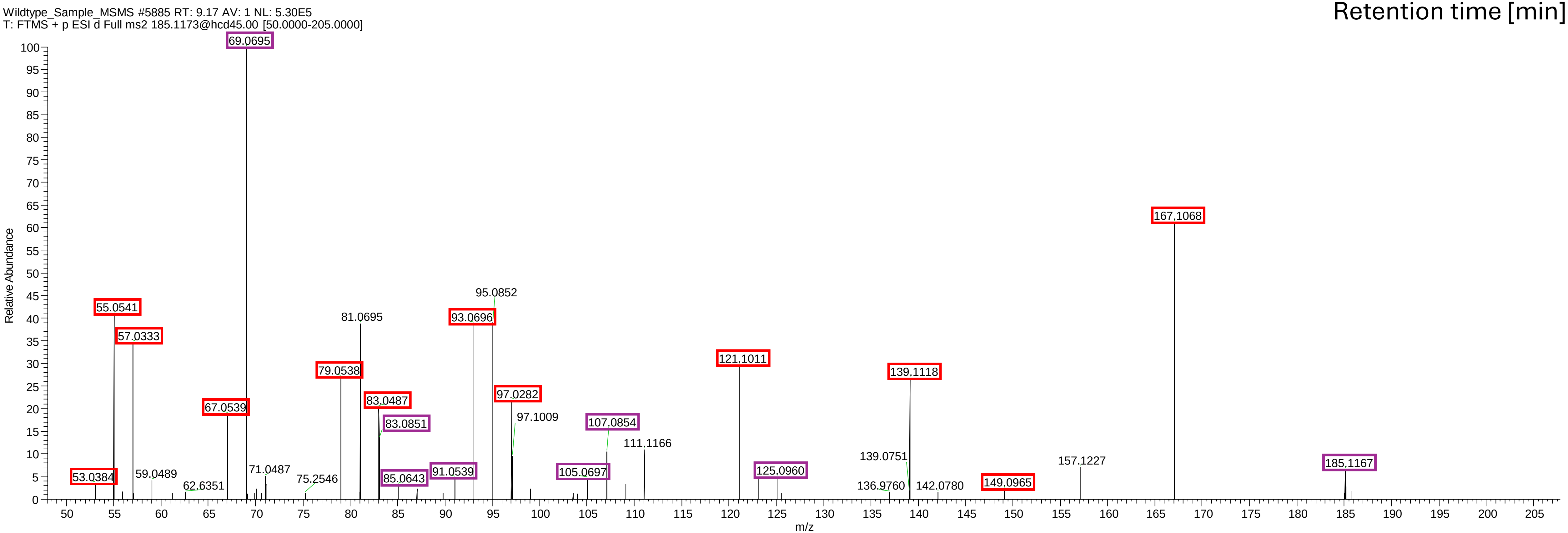

Supplement: Supplementary file 3 — Supplementary Material 3: Supplementary Figure S2 [file 12934_2025_2718_MOESM3_ESM.pdf]
